# Serotonin 5-HT_3_ Receptor-Mediated Vomiting Occurs via the Activation of Ca^2+^/CaMKII-Dependent ERK1/2 Signaling in the Least Shrew (*Cryptotis parva*)

**DOI:** 10.1371/journal.pone.0104718

**Published:** 2014-08-14

**Authors:** Weixia Zhong, Tarun E. Hutchinson, Seetha Chebolu, Nissar A. Darmani

**Affiliations:** Department of Basic Medical Sciences, College of Osteopathic Medicine of the Pacific, Western University of Health Sciences, California, United States of America; Cinvestav-IPN, Mexico

## Abstract

Stimulation of 5-HT_3_ receptors (5-HT_3_Rs) by 2-methylserotonin (2-Me-5-HT), a selective 5-HT_3_ receptor agonist, can induce vomiting. However, downstream signaling pathways for the induced emesis remain unknown. The 5-HT_3_R channel has high permeability to extracellular calcium (Ca^2+^) and upon stimulation allows increased Ca^2+^ influx. We examined the contribution of Ca^2+^/calmodulin-dependent protein kinase IIα (Ca^2+^/CaMKIIα), interaction of 5-HT_3_R with calmodulin, and extracellular signal-regulated kinase 1/2 (ERK1/2) signaling to 2-Me-5-HT-induced emesis in the least shrew. Using fluo-4 AM dye, we found that 2-Me-5-HT augments intracellular Ca^2+^ levels in brainstem slices and that the selective 5-HT_3_R antagonist palonosetron, can abolish the induced Ca^2+^ signaling. Pre-treatment of shrews with either: i) amlodipine, an antagonist of L-type Ca^2+^ channels present on the cell membrane; ii) dantrolene, an inhibitor of ryanodine receptors (RyRs) Ca^2+^-release channels located on the endoplasmic reticulum (ER); iii) a combination of their less-effective doses; or iv) inhibitors of CaMKII (KN93) and ERK1/2 (PD98059); dose-dependently suppressed emesis caused by 2-Me-5-HT. Administration of 2-Me-5-HT also significantly: i) enhanced the interaction of 5-HT_3_R with calmodulin in the brainstem as revealed by immunoprecipitation, as well as their colocalization in the area postrema (brainstem) and small intestine by immunohistochemistry; and ii) activated CaMKIIα in brainstem and in isolated enterochromaffin cells of the small intestine as shown by Western blot and immunocytochemistry. These effects were suppressed by palonosetron. 2-Me-5-HT also activated ERK1/2 in brainstem, which was abrogated by palonosetron, KN93, PD98059, amlodipine, dantrolene, or a combination of amlodipine plus dantrolene. However, blockade of ER inositol-1, 4, 5-triphosphate receptors by 2-APB, had no significant effect on the discussed behavioral and biochemical parameters. This study demonstrates that Ca^2+^ mobilization via extracellular Ca^2+^ influx through 5-HT_3_Rs/L-type Ca^2+^ channels, and intracellular Ca^2+^ release via RyRs on ER, initiate Ca^2+^-dependent sequential activation of CaMKIIα and ERK1/2, which contribute to the 5-HT_3_R-mediated, 2-Me-5-HT-evoked emesis.

## Introduction

Chemotherapy (e.g. cisplatin)-induced nausea and vomiting (CINV) is mediated via neurochemical circuits that involve brain-gut interactions [1]. The critical sites for CINV includes the medullary emetic nuclei of the dorsal vagal complex (DVC) in the brainstem, as well as the enteric nervous system (ENS) and enterochromaffin cells (EC cells) in the gastrointestinal tract (GIT) [2], [3]. The DVC emetic nuclei consists of the nucleus tractus solitarius (NTS), the dorsal motor nucleus of the vagus (DMNX) and the area postrema (AP) [1]. These brainstem emetic loci can be activated by emetogens, such as serotonin, either directly or indirectly through gastrointestinal signaling [4]. Among several, serotonin (5-hydroxytryptamine = 5-HT) is one important emetic neurotransmitter in both the brainstem and the gastrointestinal tract (GIT) that contributes to induction of CINV. In the GIT 5-HT is mainly produced and stored in the enterochromaffin (EC) cells and its release is regulated by the ENS as well as by multiple receptors present on EC cells including serotonergic 5-HT_3_ receptors (5-HT_3_Rs) [3], [5], [6]. The diverse functions associated with 5-HT are due to the existence of a large family of serotonergic receptors, 5-HT_1_ to 5-HT_7_, in which each class consist of further subtypes [7]. Unlike most serotonergic receptors which are G-protein-coupled, the 5-HT_3_R belongs to the ligand-gated ion channel receptor superfamily and is associated with vomiting. 5-HT_3_Rs are found throughout the brainstem DVC and GIT [1], [8]. In fact, cisplatin-like drugs cause vomiting via release of 5-HT from the gastrointestinal EC cells which subsequently activates local 5-HT_3_Rs present on the GIT vagal afferents [1], [9], [10]. This activation results in vagal nerve depolarization which subsequently triggers the brainstem DVC emetic nuclei to initiate the vomiting reflex.

The central/peripheral-acting agent 2-Methyl serotonin (2-Me-5-HT) is considered a “more selective” 5-HT_3_R agonist, which causes vomiting in several species including the least shrew [11], [12], [13]. In fact 2-Me-5-HT-induced emesis has been shown to be associated with enhanced Fos-immunoreactivity in both the DVC emetic nuclei and in the ENS of the least shrew [14]. Moreover, 5-HT_3_R-selective antagonists such as tropisetron [10] or palonosetron [15], can suppress vomiting caused by 2-Me-5-HT. However, to date, the downstream signaling pathways for the 5-HT_3_R-mediated vomiting remain unknown. Recently, it has been demonstrated that increased luminal glucose levels result in 5-HT release from EC cells, which subsequently activates vagal afferent 5-HT_3_Rs, leading to activation of the Ca^2+^/calmodulin-dependent kinase II (CaMKII) signaling pathway in the brainstem DVC-gut circuit in rats [16]. Activation of the extracellular signal-regulated kinase 1/2 (ERK1/2) also appears to be involved in some downstream functions of 5-HT_3_Rs including pain [17] and cisplatin-induced immediate and delayed emesis [18].

In the present study we sought to evaluate the potential involvement of the above-discussed transduction signals downstream of 5-HT_3_Rs in the process of vomiting via the use of *in vivo* pharmacology, *ex-vivo* and/or *in vitro* immunoprecipitation, immunohistochemistry, immunocytochemistry and Western blot on isolated EC cells and/or tissues of both small intestine and brainstem in the least shrew.

## Materials and Methods

### Animals and Ethics statement

Adult least shrews were bred in the animal facility of Western University of Health Sciences. Previous studies had demonstrated no gender differences, so both males and females were used. Shrews were housed in groups of 5–10 on a 14∶10 light:dark cycle, fed with food and water *ad libitum* as described previously [19]. All the shrews used were 45–60 days old and weighed between 4–5 g. This study was carried out in strict accordance with the recommendations in the guide for the Care and Use of Laboratory Animals of the National Institutes of Health (Department of Health and Human Services Publication, revised, 1985). The protocol was approved by the Western University of Health Sciences IACUC. To minimize the suffering of laboratory animals, the number of pharmacological tests was limited to the necessary minimum and the animals were observed regularly for any signs of unnecessary suffering from drug treatment. Any animal showing at least one of the following symptoms: weight loss greater than 20% of the initial weight, not eating or drinking, rough appearance of fur and/or absence of activity, were euthanized via exposure to 32% isoflurane. All experiments were conducted between 9∶00 and 15∶00 h.

### Drugs

2-Methylserotonin maleate salt (2-Me-5-HT) was purchased from Sigma/RBI (St. Louis, MO). Palonosetron was a generous gift from Helsinn Health Care (Lugano, Switzerland). The 5-HT_2A_ receptor antagonist SR46349B was purchased from Sanofi (Bridgewater, NJ). The 5-HT_6_ receptor antagonists Ro-046790 and Ro-4368554 were purchased from Sigma/RBI (St. Louis, MO) and Tocris (Minneapolis, MN), respectively. The CaMKII inhibitor KN93 and its inactive analog KN92 as well as ERK1/2 inhibitor PD98059 were obtained from Calbiochem (San Diego, CA). The L-type Ca^2+^ channel antagonist amlodipine besylate was purchased from Tocris (Minneapolis, MN). The ryanodine receptor antagonist dantrolene (sodium salt) and the inositol-1, 4, 5-triphosphate receptor antagonist 2-APB, were obtained from Santa Cruz Biotechnology (Dallas, TX). Unless otherwise stated, the above drugs were dissolved in water. KN92, dantrolene sodium and 2-APB were dissolved in 25% DMSO in water. PD98059 was dissolved in 0.5% ethanol, 0.5% Tween-80 in saline. All drugs were administered at a volume of 0.1 ml/10 g of body weight.

### Ca^2+^ imaging

#### Least shrew brainstem slice preparation and treatment

Adult least shrews were anesthetized in lethal isoflurane chamber and subsequently decapitated. Brainstems were quickly removed and transferred to ice-cold artificial cerebrospinal fluid (aCSF, pH 7.37) buffer, containing (in mM): 124 NaCl; 5 KCl; 1.3 MgCl_2_; 2 CaCl_2_; 10 glucose; and 26.2 NaHCO_3_, and aerated with 95% O_2_/5% CO_2_. Transverse brainstem slices (200 µm-thick) containing the DVC emetic nuclei identified as previously reported in our lab [14] were prepared using a Leica vibratome (Model-VT 100 A), maintained in aCSF buffer, and incubated with Ca^2+^ indicator fluo-4 AM (5 µM; Invitrogen) for 30 min in dark at room temperature. The fluo-4 AM loaded slices were pinned to sylgard blocks (Ellsworth Adhesives, Germantown, WI) and pre-treated with either the selective 5-HT_3_R antagonist palonosetron (1 µM) or its vehicle (control) for 30 min. The pretreated slices were simultaneously placed in an open bath imaging chamber (Warner Instruments, Hamden, CT) containing aCSF and mounted on the confocal imaging stage assembled with model 710 NLO (Carl Zeiss Microscopy, Thornwood, NY) laser scanning confocal imaging workstation with inverted microscope (Olympus IX81 or Zeiss Axio Observer Z1). Since only 1 section (200 µm-thick) containing the emetic nuclei could be prepared from each shrew brainstem, one slice from 4 different shrews were used to investigate the effect of palonosetron on 2-Me-5-HT-elicited Ca^2+^ increase in the AP region among the brainstem DVC emetic nuclei. 2-Me-5-HT (1 µM) was added to aCSF containing palonosetron or vehicle at the end of pretreatment using a hand pipette, exactly at the 400^th^ sec during the whole 1200-sec Ca^2+^ image-acquisition period.

#### Measurement of intracellular Ca^2+^

Slices were illuminated at 488 nm with a krypton argon laser and the emitted light was collected using a photomultiplier tube. Line scans were imaged at rates from 422 to 822 lines generated every 1 s, depending on line length. To ensure that sparks within the region of interest (ROI), the AP region of the brainstem, were imaged, global Ca^2+^ responses were acquired at roughly one image per second with an imaging depth of 10 µm, which is equivalent to two or three cells thick. The sampling depth was 16-bit (Zeiss 710). Ca^2+^ spark recordings were made using Zeiss C-Apochromat 63x/1.20 water immersion objective. ROIs were examined *post hoc* and analyzed with ImageJ. Analysis of time series recordings was achieved by hand using the time series analyzer plugin for ImageJ. For presentation purposes, the fractional fluorescence intensity was calculated as F/F0. After Ca^2+^ image acquisition, the data was analyzed by NIH-approved Fiji ImageJ software using the time series analyzer plugin for ImageJ. The captured images were visualized and cells with different level (500–60000) of fluorescence intensities were identified. Regions of interest were selected from the initial frame captured at 0 sec with cells showing initial fluorescence intensities between 5000–25000 and the values of fluorescence intensities at different time points were identified by time series analyzer to plot the graphs of selected regions of interest. To show the changes in Ca^2+^ levels before and after 2-Me-5-HT treatment, the average fluorescence intensities were calculated for at least 12 regions of interest in each acquisition for all time points. The data is represented in a graph as the ratio (F/F0) of final fluorescence intensity (F) for each time point to the initial fluorescence intensities (F0) at 0 sec for ROIs and is the mean value of 4 individual experiments.

### Behavioral emesis studies

On the day of the experiment shrews were brought from the animal facility, separated into individual cages and allowed to adapt for at least two hours (h). Daily food was withheld 2 h prior to the start of the experiment but shrews were given 4 mealworms each prior to emetogen injection, to aid in identifying wet vomits as described previously [20].

We have previously demonstrated that a 5 mg/kg intraperitoneal (i.p.) injection of 2-Me-5-HT produces a robust frequency of vomits in all tested animals [13], [18]. To evaluate whether pretreatment with inhibitors/antagonists may affect the frequency of emesis and/or percentage of shrews vomiting in response to 2-Me-5-HT administration, different groups of shrews were pre-treated with an injection of either corresponding vehicle (i.p. or subcutaneous (s.c.)), or varying doses of the: i) L-type Ca^2+^ channel antagonist amlodipine (1, 5, and 10 mg/kg, s.c., n = 6–8 per group); ii) ryanodine receptor (RyRs) antagonist dantrolene (1, 5, 10, and 20 mg/kg, i.p., n = 6–11 per group); iii) inositol-1,4,5 triphosphate (IP_3_) receptor antagonist 2-APB (0.25, 1, 5 and 10 mg/kg, i.p., n = 6 per group); iv) serotonin 5-HT_2A_ receptor antagonist SR46349B (5 and 10 mg/kg, s.c., n = 5–6 per group); v) serotonin 5-HT_6_ receptor antagonists Ro-046970 or Ro-4368554 (0.25, 1, 5, 10 and 20 mg/kg, i.p., n = 5–6 per group), vi) active inhibitor of CaMKII KN93 (2.5, 5 and 10 mg/kg, i.p., n = 6–8 per group); vii) inactive analog of KN93, KN92 (10 mg/kg, i.p., n = 6); or viii) ERK1/2 inhibitor PD98059 (2.5 and 5 mg/kg, i.p., n = 6–8 per group). Thirty minutes later, each treated shrew received a 5 mg/kg emetic dose of 2-Me-5-HT (i.p.). The number of animals vomiting within groups and the frequency of vomits for the next 30 min were recorded. The antiemetic effects of a combination of semi-active doses of amlodipine (s.c.) with dantrolene (i.p.) were further investigated. Thus, different groups of shrews (n = 8–11 per group) were pre-treated either with their corresponding vehicles (Aml 0 + Dan 0), amlodipine 5 mg/kg + dantrolene vehicle (Aml 5 + Dan 0), amlodipine vehicle + dantrolene 10 mg/kg (Aml 0 + Dan 10), or amlodipine 5 mg/kg + dantrolene 10 mg/kg (Aml 5 + Dan 10), 30 min prior to 2-Me-5-HT administration. The indices of induced emesis were recorded as described above. Each shrew was used once and then euthanized with an overdose of pentobarbital (100 mg/kg, i.p.) following the termination of each experiment.

### Tissue studies

#### Tissue collection

Adult least shrews treated with 2-Me-5-HT (5 mg/kg, i.p.) were rapidly anesthetized with isoflurane and decapitated at the indicated time points post-treatment (see Figures). Brainstem and small intestine were quickly removed. Further division of the small intestine to recognize the jejunal segment was performed according to Ray et al [7]. Brainstem and jejunum were transferred into cold fixative 4% paraformaldehyde (PF) in phosphate-buffered saline (PBS) for cryo-sectioning and immunohistochemical staining. For biochemical assays, the lower half of brainstem, mostly medullary structures, was isolated and immediately frozen on dry ice.

#### Immunohistochemistry

The optimal-cutting-temperature compound-embedded brainstems (n = 3 animals per group) were cut into 20 µm sections using a cryostat and mounted onto slides. Sections from brainstem were observed with a light microscope and those containing the whole DVC subjected to immunohistochemistry. Slides were washed in PBS three times, fixed with 4% PF for 2 h at 4°C, then washed 3 times with PBS, permeabilized with 0.1% Triton X-100 for 30 min at 4°C, and washed again 3 times with PBS. After blockade for 1 h with the blocking buffer containing 5% bovine serum albumin (BSA) in PBS, histological sections of brainstem and intestine were co-incubated overnight at 4°C with goat anti-CaMKIIα (1∶100, ab87597, Abcam) and rabbit anti-phospho-CaMKIIα (Thr286) antibodies (1∶100, ab5683, Abcam) to analyze CaMKIIα phosphorylation at Thr286 site. Sections were then washed 3 times (10 min each) in PBS and incubated in Alexa Fluor 594 donkey anti-goat IgG and Alexa Fluor 488 donkey anti-rabbbit IgG (1∶400, Abcam) for 2 h at room temperature. Images for the whole DVC region and for the individual areas (AP/NTS/DMNX) were acquired under a confocal microscope (Nikon) with Metamorph software using 20× and 100× objectives, respectively. Nuclei of cells were stained with DAPI. DAPI is excited at 345 nm and emits at 458 nm, producing blue fluorescence.

Immunohistochemistry was also performed to analyze 5-HT_3_R-CaM colocalization on brainstem slices (20 µm) and jejunum slices (10 µm). Sections were co-incubated overnight at 4°C with rabbit anti-5-HT_3_R (1∶100, sc-28958, Santa Cruz) and mouse anti-CaM antibodies (1∶100, MA3-917, Thermo) to analyze co-localization of 5-HT_3_R and CaM. After washing in PBS, the immunoreactivities were visualized by incubation with Alexa Fluor 594 donkey anti-rabbit IgG and Alexa Fluor 488 donkey anti-mouse IgG (1;400, Abcam) or rhodamine red anti-rabbit and FITC-conjugated anti-mouse secondary antibodies (1∶100, Jackson Immuno Research Laboratories). Images for the whole DVC region and for the individual nuclei (AP/NTS/DMNX) were acquired under a confocal microscope (Nikon) with Metamorph software using 200× objective.

#### Immunoprecipitation and Western blot

To assess the 2-Me-5-HT-induced interaction of 5-HT_3_R and CaM in the brainstem of least shrews, the animals (n = 3 per group) were treated either with vehicle, the 5-HT_3_R agonist 2-Me-5-HT (5 mg/kg, i.p.), the 5-HT_3_R antagonist palonosetron (5 mg/kg, s.c.), or a combination of both agents. The time to first vomit was generally within 15 minutes of 2-Me-5-HT injection. Thus, each shrew brainstem was isolated 20 min after 2-Me-5-HT treatment, homogenized in cold lysis buffer (50 mM Tris-HCL, pH 8, 150 mM NaCl and 1% NP-40) containing protease- and phosphatase-inhibitors cocktail (Pierce, Rockford, IL), and centrifuged at 10000×g for 20 min at 4°C. Total protein concentrations in supernatants were confirmed using BCA protein assay kit (Pierce, Rockford, IL). A 1 mg protein extract from each brain lysate was immunoprecipitated overnight at 4°C with 10 µg rabbit anti-5-HT_3_R antibody (sc-28958) or rabbit IgG (sc-2027, Santa Cruz) and then incubated with 50% Protein A/G agarose slurry (20421, Thermo) for 1 h with occasional mixing at 4°C. After washing 3 times with lysis buffer by centrifuging at 700×g for 1 min at 4°C, supernatant was discarded, and 50 µl of 1.5× SDS-PAGE sample buffer was added to the saved pellets, heated at 100°C for 10 min, and centrifuged at 700×g for 1 min. Supernatants containing 5-HT_3_R immunoprecipitates were subjected to Western blot for the detection of 5-HT_3_R and CaM. Inputs from various groups were used to confirm the expression of 5-HT_3_R and CaM. GAPDH served as an internal standard. All samples were subjected to 12% SDS-polyacrylamide gel electrophoresis. Proteins were transferred to a polyvinylidene difluoride membrane for 90 min at 90 V. After blocking with TBST solution (50 mM Tris-HCl, pH 7.5, 150 mM NaCl, and 0.1% Tween 20) containing 5% BSA for 1 h at room temperature, membranes were incubated overnight at 4°C with mouse anti-CaM antibody (1∶1000, 05–173, Millipore), goat anti-5-HT_3_R antibody (1∶500, sc-19152, Santa Cruz) or mouse anti-GAPDH antibody (1∶10000, MAB374, Chemicon). Infrared fluorescent-labeled anti-goat and anti-mouse secondary antibodies (1∶10000, LI-COR Biosciences) were then used. Bound antibodies were visualized using Odyssey imaging system and analyzed semi-quantitatively based on densitometric values using Quantity-One 1D software (Bio-Rad). The ratios of CaM (∼17 kD) to 5-HT_3_R (∼55 kD) precipitated by 5-HT_3_R antibody were calculated and are shown as fold change of control.

#### Western blot for CaMKIIα or ERK1/2 phosphorylation analyses

To determine the time-dependent profile of CaMKIIα and ERK activation, different groups of animals (n = 3 per group) were sacrificed at 5, 10, 20, 30, 60 min following 2-Me-5-HT administration (5 mg/kg, i.p.). In addition, different groups of least shrews (n = 3 per treatment group) were pretreated with either palonosetron (5 mg/kg, s.c.), SR46349B (10 mg/kg, i.p.), 2-APB (10 mg/kg, i.p.), amlodipine (10 mg/kg, s.c.), dantrolene (20 mg/kg, i.p.), amlodipine (5 mg/kg, s.c.) + dantrolene (10 mg/kg, i.p.), KN93 (10 mg/kg, i.p.), PD98059 (5 mg/kg, i.p.), or corresponding vehicles, 30 min before 2-Me-5-HT injection (5 mg/kg, i.p.). Shrew brainstems were then removed at specific intervals after 2-Me-5-HT treatment and homogenized in lysis buffer. Protein extracts from brainstem lysates were subjected to Western blot. Primary antibodies included rabbit anti-phospho-CaMKII (Thr286) (1∶1000, ab32678, Abcam), mouse anti-CaMKII (1∶1000, ab22609, Abcam), rabbit anti-phospho-ERK1/2 (1∶1000, 9101, Cell Signaling) and mouse anti-ERK1/2 (1∶3000, 9107, Cell Signaling) antibodies. Infrared fluorescent-labeled anti-rabbit or anti-mouse secondary antibodies (1∶10000, LI-COR Biosciences) were used. Bound antibodies were visualized correspondingly using Odyssey imaging system and analyzed semi-quantitatively based on densitometric values using Quantity-One 1D software (Bio-Rad). The ratios of pCaMKIIα (∼50 kD) to CaMKIIα and pERK1/2 (42/44 kD) to ERK1/2 were calculated and presented as fold change of control.

### Cellular studies

#### Isolation of enterochromaffin cells

The enterochromaffin (EC) cell isolation from naïve shrews was performed via a slight modification of the method described by Schäfermeyer and co-workers [21]. Buffers A, B and C were prepared according to Schäfermeyer and co-workers [21]. Shrew intestines were surgically removed and enzymatic digestion and alternative switching off and on exposure to EDTA-calcium salt was performed for isolation of intestinal mucosal cells. Each intestine (approximately 12 cm in length and 3mm in diameter) was fastened by a small metal binder clip at its anal end, and was filled with the buffer B (containing a mixture of 0.64 mg/ml pronase E and 0.5 mg/ml collagenase) by injecting and filling it with 1–1.5 ml from the proximal end which was then closed by a small metal binder clip to make sacs. The filled intestines were partially immersed in 100 mm plastic dishes containing 2 ml buffer B and incubated at 37°C for 15 min. The intestines were hung vertically from the distal metal binder and the proximal metal binder was then removed by cutting the intestine from its edge to release the digested, detached mucosal lining from muscularis propria. In addition, the mucosal lining was stripped from the distal to the proximal end of intestine by tweezing and running forceps along the intestinal length. The mucosal lining was collected into a petri dish containing buffer A (25 ml) for 20 min, then centrifuged at 1200 rpm for 10 min. Buffer B was added to the pellet, gently vortexed and stirred for 10 min. The EC cells were collected by pouring the mixture through a nylon filter mesh (pore size <200 µm) and buffer B (25 ml) was added and centrifuged at 1200 rpm for 10 min. Enriched EC cells were obtained by step density gradient centrifugation using nycodenz gradient with adjusted density of 1.1 g/ml at the bottom of tube, followed by adjusted density of 1.07 g/ml as intermediate layer. The cell suspension was layered on top of the two layers, centrifuged at 1100 rpm for 8 min with slow deceleration. The EC cell-enriched layer was collected at the 1.070 interface, and then washed in buffer C.

#### Cell Treatment and CaMKIIα phosphorylation analyses

The EC cells (n = 3 expeiments per group) were pre-incubated for 30 min with either 1 µM palonosetron or its vehicle at 37°C, followed by 5-HT_3_R stimulation with 1 µM 2-Me-5-HT (or its vehicle as control) for 20 min at 37°C. After treatment, the collected cell pellets were re-suspended in lysis buffer, sonicated and centrifuged (10000×g, 10 min at 4°C). Supernatants containing total protein were quantified and used to analyze CaMKIIα phosphorylation at Thr286 by Western blot, as described above. For immunocytochemistry, control and treated EC cells (n = 3 experiments per group) were fixed with 4% PF in PBS for 10 min followed by treatment with cold methanol for 10 min at 4°C. The cells were also co-stained with rabbit anti-CaMKIIα (1∶100, sc-13082, Santa Cruz) and mouse anti-phospho-CaMKIIα (Thr286) (1∶100, sc-32289, Santa Cruz). The immunoreactivities were visualized by incubation with rhodamine red anti-rabbit and FITC-conjugated anti-mouse antibodies.

### Statistical analysis

The vomit frequency data were analyzed using the Kruskal-Wallis non-parametric one-way analysis of variance (ANOVA) and followed by Dunn’s *post hoc* test. The percentage of animals vomiting across groups at different doses was compared using the chi square test. Statistical significance for differences between two groups was tested by unpaired t-test, among groups (≥3) was tested by one-way ANOVA followed by Tukey’s multiple comparison tests. For time-course analyses of CaMKIIα and ERK activation, one-way ANOVA followed by Dunn’s post hoc test was used. All results are presented as mean ± SEM. P<0.05 was considered significant.

## Results

### 5-HT_3_R stimulation increases intracellular Ca^2+^ concentration and Ca^2+^ mobilization regulates 2-Me-5-HT-induced emesis

Activation of 5-HT_3_Rs regulates neuronal function by directly gating its corresponding ion channel to produce an increase in Ca^2+^ influx which rapidly induces neuronal depolarization [22]. In addition, the increase in the magnitude of the intracellular Ca^2+^ signal can be partly due to subsequent extracellular Ca^2+^ influx via enhancement of voltage-operated Ca^2+^ channels [23] due to mobilization of intracellular Ca^2+^ from ER stores through the process of Ca^2+^-induced Ca^2+^ release (CICR) [24].

**Figure 1:**
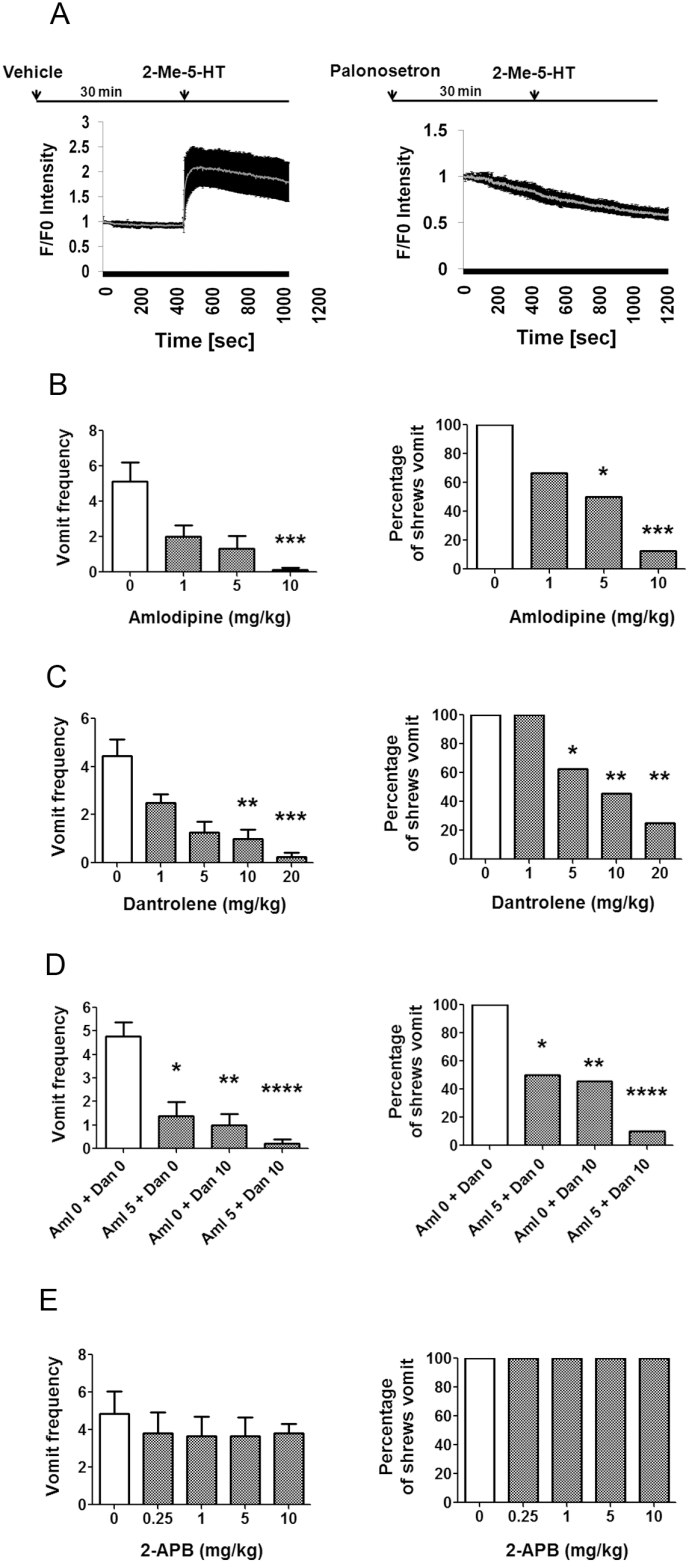
Effects of prior administration of extracellular and intracellular Ca^2+^ antagonists on emesis induced by the 5-HT_3_R agonist 2-Me-5-HT, which evokes Ca^2+^ responses. Graph A) Increased intracellular Ca^2+^ levels *(*as demonstrated by fluo-4 AM) caused by the selective 5-HT_3_R agonist, 2-Me-5-HT (1 µM), in the least shrew brainstem area postrema (AP) region in the absence (vehicle, left panel) and presence of the selective 5-HT_3_R antagonist, palonosetron (1 µM) (right panel). Graphs B–E) Effects of Ca^2+^ modulators on the frequency and percentage of shrews vomiting in response to 2-Me-5-HT administration (5 mg/kg, i.p.). Different groups of least shrews were given an injection of either the corresponding vehicle, or varying doses of: 1) the L-type Ca^2+^ channel blocker, amlodipine (s.c.) (B); 2) the ryanodine receptor antagonist, dantrolene (i.p.) (C); 3) lower but combined doses of amlodipine (Aml, 5 mg/kg, s.c.) plus dantrolene (Dan, 10 mg/kg, i.p.) (D); or 4) the inositol-1, 4, 5-triphosphate receptor blocker, 2-APB (i.p.) (E); which were administered 30 min prior to 2-Me-5-HT injection. For each case, the vomiting responses were recorded for 30 min post 2-Me-5-HT administration. The frequency data is presented as mean ± SEM. *P<0.05, **P<0.01, ***P<0.001 and ****P<0.0001 compared with corresponding vehicle-pretreated controls.

Here, to explore the signaling pathway for 5-HT_3_R-mediated emesis, changes in intracellular Ca^2+^ signaling were first examined. Thus, incubation of isolated least shrew brainstem slices containing the DVC emetic loci with the selective 5-HT_3_R agonist 2-Me-5-HT (1 µM) resulted in a rapid increase in intracellular Ca^2+^ concentration monitored via an increase in fluo-4 AM fluorescence intensity, as shown by the increased F/F_0_ ratio (Figure 1A, left panel). Indeed, following addition of 2-Me-5-HT, intracellular Ca^2+^ levels reached maximum rapidly in 100 seconds which then declined without full recovery within the remaining recording period. Blockade of 5-HT_3_Rs in brainstem slices by the selective 5-HT_3_R antagonist palonosetron (1 µM) slightly reduced the baseline Ca^2+^ levels and fully suppressed the 2-Me-5-HT-induced enhancement of intracellular Ca^2+^ signaling (Figure 1A, right panel).

We then addressed the relevance of 5-HT_3_R-mediated Ca^2+^ influx in the anti-emetic potential of L-type Ca^2+^ channel blockers on vomiting caused by the selective 5-HT_3_R agonist 2-Me-5-HT. Administration of 2-Me-5-HT (5 mg/kg, i.p.) elicited vomiting in all the tested shrews (Figure 1B–E). Pretreatment with the L-type Ca^2+^ channel blocker amlodipine (0, 1, 5, or 10 mg/kg, s.c.) dose-dependently suppressed both the vomit frequency (KW (3, 23) = 14.77, P<0.01) (Figure 1B, left panel) and the percentage of shrews vomiting (χ^2^ (3, 23) = 11.98; P<0.01) in response to 2-Me-5-HT (Figure 1B, right panel). In fact a significant reduction in vomit frequency occurred at 10 mg/kg (P<0.001), whereas substantial reductions in the percentage of shrews vomiting were seen at 5 (P<0.05) and 10 mg/kg (P<0.001) doses. We next investigated whether Ca^2+^ release from the ER via ryanodine receptors (RyRs) and/or inositol-1, 4, 5-triphosphate receptors (IP_3_Rs), were involved in 2-Me-5-HT-induced vomiting. Administration of dantrolene (1, 5, 10, 20 mg/kg, i.p.), a blocker of RyRs, dose-dependently suppressed both the 2-Me-5-HT-induced vomit frequency (KW (4, 37) = 23.35, P<0.001) and the percentage (χ^2^ (4, 37) = 15.42, P<0.01) of shrews vomiting with significant reductions occurring at 5, 10 and 20 mg/kg doses (Figure 1C, P<0.05, P<0.01 and P<0.01, respectively). Moreover, a near complete blockade in both emetic parameters was attained (KW (3, 34) = 20.88, P<0.001) and (χ^2^ (3, 34) = 15.49, P<0.01, respectively) in shrews pretreated with lower but combined doses of amlodipine (5 mg/kg) plus dantrolene (10 mg/kg) (Figure 1D). In contrast, blockade of IP_3_Rs with 2-APB (0.25, 1, 5, and 10 mg/kg) had no effect on 2-Me-5-HT-evoked vomiting responses (Figure 1E). These behavioral results suggest that extracellular Ca^2+^ influx through Ca^2+^ channels in plasma membrane and subsequent release of Ca^2+^ from the dantrolene-sensitive intracellular ER Ca^2+^ channels, RyRs, play significant roles in the mediation of the vomiting caused by 2-Me-5-HT.

### 2-Me-5-HT enhances interaction of 5-HT_3_R with CaM in the brainstem of least shrews

5-HT_3_R stimulation induces extracellular Ca^2+^ influx which may secondarily affect the cytosolic Ca^2+^ sensor protein, calmodulin (CaM), since an increase in free cytoplasmic Ca^2+^ concentration can lead to activation of CaM and CaMKIIα [25]. CaM can bind a number of other targets including enzymes, ion channels, transcription factors and several plasma membrane receptors [26]. CaM not only can modulate G-protein-coupled receptor signaling including serotonergic 5-HT_1A_-, 5-HT_2A_- and 5-HT_2C_-recptors [27], [28], [29], but may also regulate the actions of diverse ion channels such as voltage-gated L-type Ca^2+^ channels, voltage-gated sodium channels and voltage-gated potassium channels [30], [31], [32].

**Figure 2:**
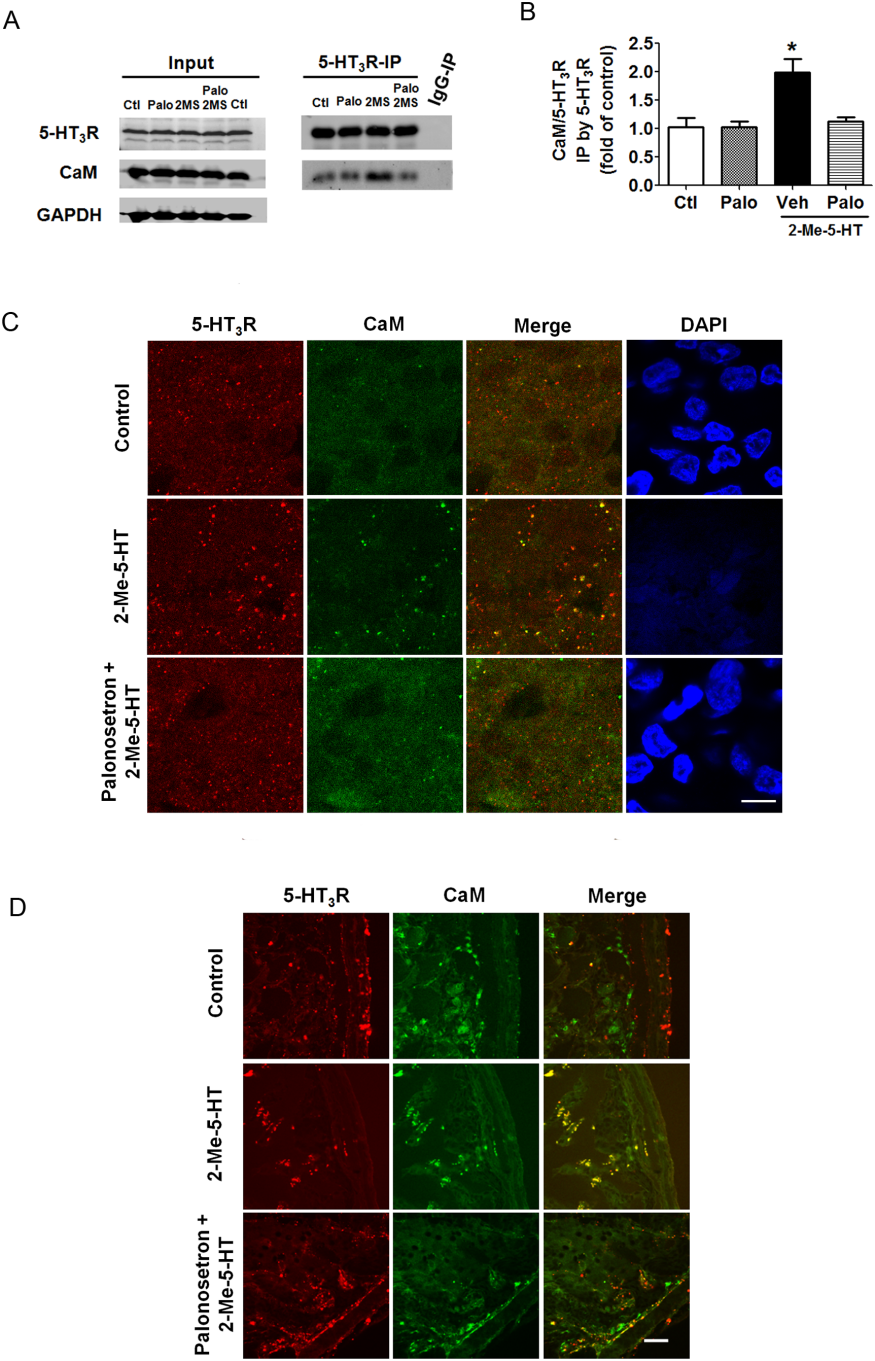
2-Me-5-HT enhances 5-HT_3_R-calmodulin (CaM) colocalization in a palonosetron-sensitive manner in least shrew brainstem and intestine. Graphs A and B) Effects of the 5-HT_3_R agonist 2-Me-5-HT and the 5-HT_3_R antagonist palonosetron on 5-HT_3_R-CaM interaction in the least shrew brainstem as revealed by co-immunoprecipitation (IP). Palonosetron (Palo, 5 mg/kg, s.c) or its vehicle (Veh) was administered 30 min prior to 2-Me-5-HT (or its vehicle) in different groups of shrews. Twenty minutes following 2-Me-5-HT administration (5 mg/kg, i.p.), brainstems were collected from the Control (Ctl) group (Veh + Veh), 2-Me-5-HT group (Veh + 2-Me-5-HT), Palonosetron group (Palo + Veh) and Palonosetron + 2-Me-5-HT group (Palo + 2-Me-5-HT). Proteins were immunoprecipitated by rabbit anti-5-HT_3_R antibody and Western blots were developed on 5-HT_3_R immunoprecipitates using goat anti-5-HT_3_R antibody and mouse anti-CaM antibody. The ratio of optical density for CaM (17 kD) to 5-HT_3_R (55 kD) was acquired and expressed as fold change of control. A) The representative Western blot, and B) Summarized data. *P<0.05 vs. the Control. Graphs C and D show the immunohistochemical analysis of 5-HT_3_R-CaM colocalization in brainstem (C) and intestinal slices (D) from shrews treated as described for A and B. 10 µm thick cryo-sections of brainstem and intestine were co-labeled with rabbit anti-5-HT_3_R and mouse anti-CaM antibodies. Representative high magnification fluorescence images (200×) show colocalization of 5-HT_3_R and CaM in the area postrema (AP) region of brainstem (C) and jejual segment of intestine (D) which were increased following 5-HT_3_R stimulation by 2-Me-5HT (5 mg/kg, i.p.). A 30 min prior exposure to the 5-HT_3_R antagonist palonosetron (5 mg/kg, s.c.) abolished the 2-Me-5-HT-induced enhancement of the 5-HT_3_R-CaM colocalization. Scale bar, 10 µm.

To determine the regulation of CaM following 5-HT_3_R activation, we investigated the interaction of CaM with 5-HT_3_R in the least shrew brainstem via co-immunoprecipitation. We have previously demonstrated that the 5-HT_3_R antagonist, palonosetron, dose-dependently suppresses vomiting evoked by 2-Me-5-HT with approximately 70% maximal protection at 5 mg/kg [15]. In the latter study we noticed that the duration of 2-Me-5-HT-induced emetic activity may range from 3–25 min post-injection in least shrews, hence a 20 min agonist exposure was chosen. Thus, subsequent to 2-Me-5-HT administration (5 mg/kg, i.p.), the shrew brainstems of different treatment groups [i.e. control (pretreated with palonosetron vehicle 30 min prior to 2-Me-5-HT vehicle injection); 2-Me-5-HT (pretreated with palonosetron vehicle 30 min prior to 2-Me-5-HT injection); palonosetron (pretreated with palonosetron (5 mg/kg, s.c.) 30 min prior to 2-Me-5-HT vehicle injection); palonosetron + 2-Me-5-HT (pretreated with palonosetron (5 mg/kg, s.c.) 30 min before injection with 2-Me-5-HT)] were collected. Proteins extracted from the brainstems were immunoprecipitated by 5-HT_3_R antibody. The resulting 5-HT_3_R immunoprecipitates were used to detect 5-HT_3_R and CaM. As shown in Figure 2A and 2B, 2-Me-5-HT elevated the interaction between 5-HT_3_Rs and CaM (P<0.05 vs. control), whereas following 5-HT_3_R blockade with palonosetron, 2-Me-5-HT failed to increase the interaction of 5-HT_3_R with CaM (P>0.05 vs. control). As with palonosetron, amlodipine suppressed the interaction of 5-HT_3_R-CaM (data not shown).

We further investigated the colocalization of 5-HT_3_R with CaM in brainstem in response to 2-Me-5-HT treatment by immunohistochemistry. Brainstems from the above-discussed experimental shrews were isolated, sections were prepared and immunolabeled for 5-HT_3_R and CaM. The colocalization between 5-HT_3_R and CaM in different DVC emetic loci in the brainstem (NTS, DMNX, and AP) were then evaluated. Brainstem sections obtained from the 2-Me-5-HT-treated shrews exhibited significantly enhanced 5-HT_3_R-CaM colocalization in AP area relative to vehicle control, whereas the brainstem sections obtained from least shrews pretreated with palonosetron followed by 2-Me-5-HT (i.e. palonosetron + 2-Me-5-HT) did not show significant alteration in 5-HT_3_R-CaM colocalization, which was similar to control (Figure 2C). However, 5-HT_3_R activation with 2-Me-5-HT had no major effect on 5-HT_3_R-CaM colocalization in NTS and DMNX (Figure S1). The above results indicate that activation of 5-HT_3_Rs can lead to the close physical connection between 5-HT_3_R and CaM in AP emetic region of the brainstem.

### 2-Me-5-HT enhances colocalization of 5-HT_3_R with CaM in the GIT of least shrews

Since the GIT plays a major role in vomiting and Darmani et al. [1] have previously demonstrated that largest increases in jejunal 5-HT tissue levels were closely associated with cisplatin-induced peak early and delayed vomit frequency, the colocalization between 5-HT_3_R and CaM in the shrew jejunum after administration of 2-Me-5-HT was also analyzed by immunohistochemistry (Figure 2D). After a 20-min exposure to 2-Me-5-HT, the least shrew intestines (excluding colon and stomach) were dissected from vehicle/vehicle-treated control, vehicle/2-Me-5-HT, and palonosetron + 2-Me-5HT treatment groups. Transverse sections were prepared from jejunum. The cryosections were immunolabeled for 5-HT_3_R and CaM and intestinal mucosal cells from jejunal regions were analyzed for interaction of 5-HT_3_R with CaM. As shown in Figure 2D, relative to the control group, the jejunal section from least shrews treated with 2-Me-5-HT exhibited significantly enhanced 5-HT_3_R-CaM colocalization. On the other hand, the jejunal sections obtained from least shrews pretreated with palonosetron followed by 2-Me-5-HT injection, showed no significant change in 5-HT_3_R-CaM colocalization and were essentially identical to the vehicle control. These results demonstrate that 2-Me-5-HT induces a 5-HT_3_R-mediated increase in 5-HT_3_R-CaM colocalization in the jejunum of the least shrew intestine.

### Activation of CaMKIIα by 2-Me-5-HT in brainstem of least shrews occurs via 5-HT_3_Rs

**Figure 3:**
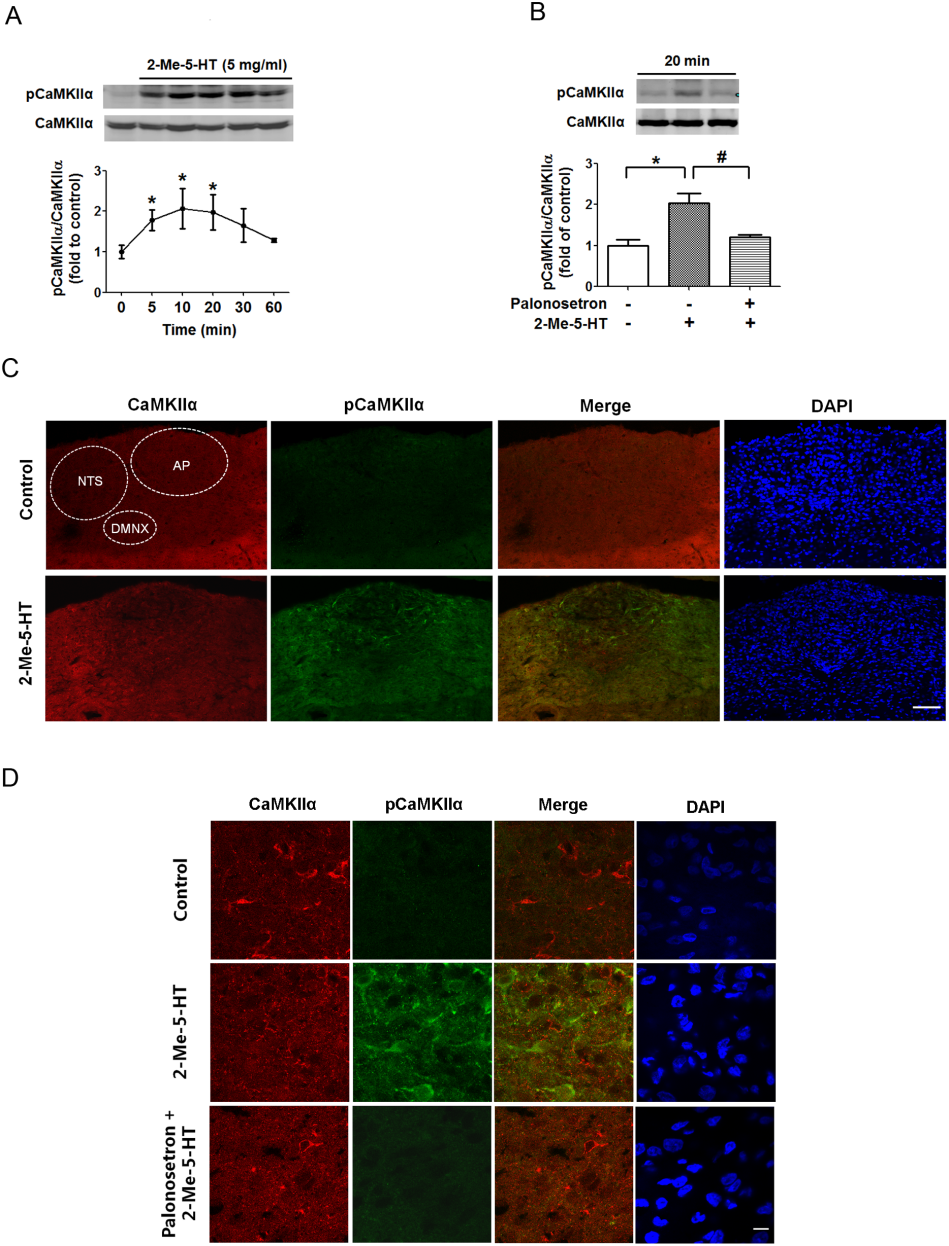
Palonosetron suppresses the ability of 2-Me-5-HT to increase CaMKIIα phosphorylation in the least shrew brainstem. A) The time-course of 2-Me-5-HT-induced CaMKIIα activation in the least shrew brainstem. Shrews were injected with the 5-HT_3_R agonist 2-Me-5-HT (5 mg/kg, i.p.) and brainstems were collected at 5, 10, 20, 30 and 60 min. Phosphorylated CaMKIIα at Thr286 (pCaMKIIα) and total CaMKIIα of samples from individual animals were determined by immunoblot with rabbit anti-pCaMKIIα and mouse anti-CaMKIIα antibodies. The ratios of pCaMKIIα (∼50 kD) to CaMKIIα were calculated and expressed as fold change of vehicle-treated controls (0 min). n = 3 per group. *P<0.05 vs. 0 min. Graph A shows the summarized data and the insets exhibit the representative Western blot. B) Palonosetron (5 mg/kg, s.c.) or its vehicle was given 30 min before 2-Me-5-HT. Immunoblots were performed on the brainstems of the least shrews sacrificed 20 min after 2-Me-5-HT administration using anti-pCaMKIIα and CaMKIIα antibodies. n = 3 per treatment group. *P<0.05 vs. vehicle/vehicle control. ^#^P<0.05 vs. vehicle + 2-Me-5-HT. Graph B displays the summarized data and the insets show the representative Western blot. C) Representative low magnification (20×) images for the brainstem dorsal vagal complex (DVC) emetic nuclei including the area postrema (AP), the nucleus tractus solitarius (NTS) and the dorsal motor nucleus of the vagus (DMNX) from sections co-labeled with rabbit anti-CaMKIIα (red) and mouse anti-pCaMKIIα (green) antibodies. Shrews were sacrificed 20 min after vehicle or 2-Me-5-HT administration. Scale bar, 100 µm. D) Representative images of high magnification (100×) showed 5-HT_3_R-mediated CaMKIIα activation in brainstem AP area. Scale bar, 10 µm.

CaMKIIα is a downstream kinase which is activated by Ca^2+^/CaM signaling, and integrates transient, localized changes in intracellular Ca^2+^ levels to induce diverse downstream responses [25], [33]. To determine the involvement of CaMKIIα in 2-Me-5-HT-induced emesis, we performed Western blots to analyze the degree of activation by CaMKIIα autophosphorylation at Thr286 (pCaMKIIα) on protein samples extracted from brainstems of 2-Me-5-HT-treated shrews. To determine the time-dependent profile of CaMKIIα activation, different groups of animals were sacrificed at 5, 10, 20, 30, 60 min following 2-Me-5-HT administration (5 mg/kg, i.p.). The results demonstrate that compared with 0 min (vehicle control), pCaMKIIα was significantly increased at 5 min (P<0.05), peaked at 10 min (P<0.05) and remained elevated up to 20 min (P<0.05) following 2-Me-5-HT injection (Figure 3A). To investigate whether pretreatment (30 min) with the 5-HT_3_R antagonist palonosetron (5 mg/kg, s,c.) can inhibit the 2-Me-5-HT (5 mg/kg, i.p.)-induced CaMKIIα activation, the tested animals were sacrificed 20 min after 2-Me-5-HT administration. The results revealed that palonosetron pretreatment abolished the 2-Me-5-HT-evoked increase in pCaMKIIα (P<0.05 vehicle + 2-Me-5-HT vs. vehicle/vehicle control; P<0.05 palonosetron + 2-Me-5-HT vs. vehicle + 2-Me-5-HT) (Figure 3B).

We further confirmed our Western blot results with immunohistochemistry (Figure 3C). Brainstem sections from vehicle control, 2-Me-5-HT and palonosetron + 2-Me-5-HT groups were prepared, and co-stained with anti-CaMKIIα and anti-phospho-CaMKIIα Thr286 antibodies. In figure 3C, immunolabeling for CaMKIIα in the control brainstem section indicated the cytoarchitectonic differences among the AP, NTS and DMNX under low magnification (20×). Immunoreactive brainstem sections showed that systemic administration of 2-Me-5-HT induced a significant increase in CaMKIIα phosphorylation at Thr286 (pCaMKIIα) throughout the DVC including AP, NTS and DMNX, but especially in AP region of least shrew brainstem (Figure 3C; Figure S2). Pre-treatment with palonosetron significantly suppressed the pCaMKIIα increase in the AP region of shrew brainstem in response to 2-Me-5-HT (Figure 3D).

### 2-Me-5-HT induces CaMKIIα activation via 5-HT_3_Rs in the EC cells in vitro

**Figure 4:**
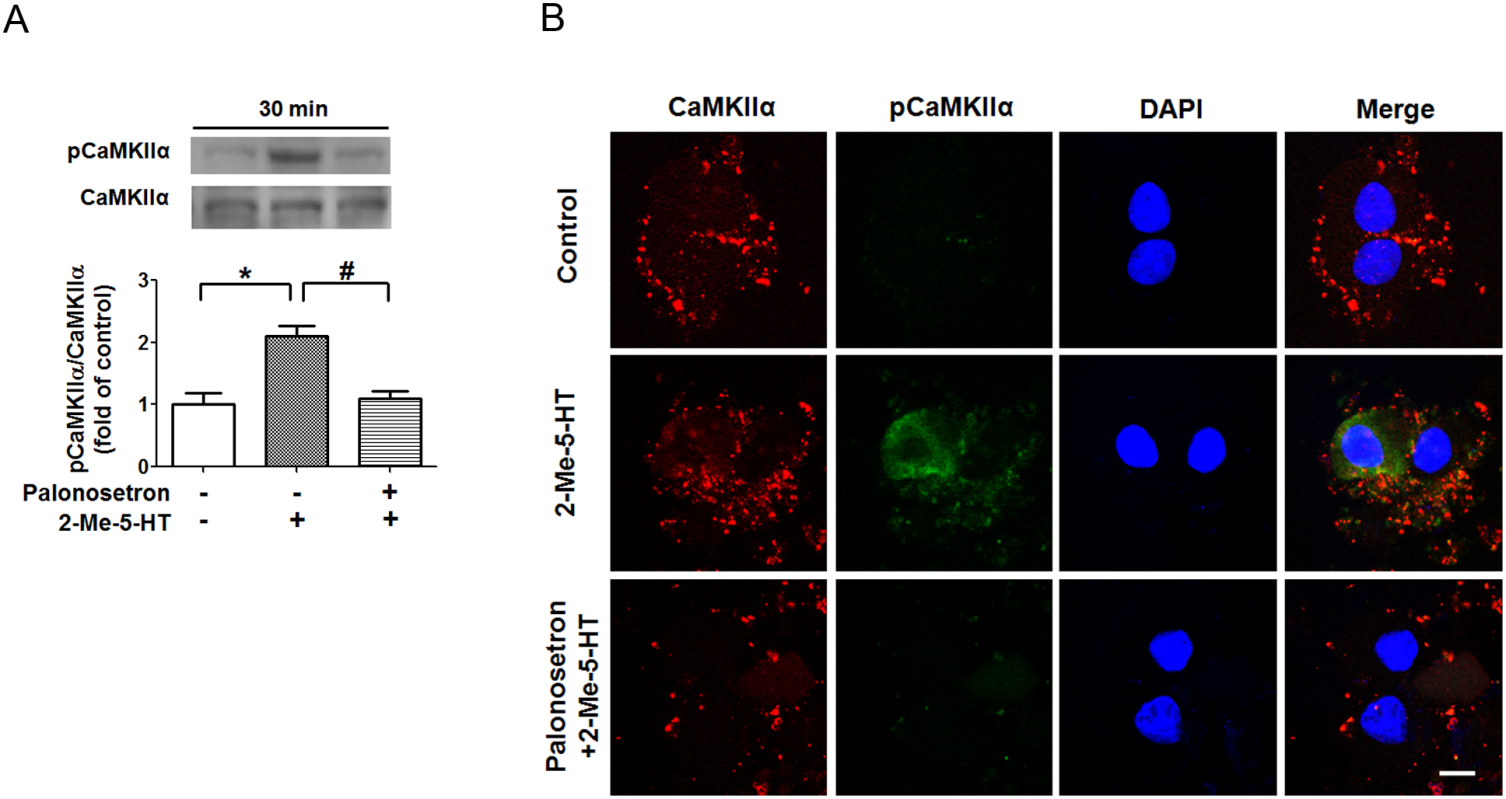
Palonosetron suppresses the ability of 2-Me-5-HT to upregulate CaMKIIα phosphorylation in enterochromaffin (EC) cells. The isolated EC cells from the least shrew intestine were incubated with the 5-HT_3_R antagonist palonosetron (1 µM) or its vehicle for 30 min and then the 5-HT_3_R agonist 2-Me-5-HT (1 µM) was added for the next 30 min. The corresponding antagonist and agonist vehicles were also incubated with EC cells and were used as control. A) The control and treated EC cells were harvested to analyze CaMKIIα phosphorylation (Thr286) using Western blot. n = 3 experiments per treatment group. *P<0.05 vs. vehicle/vehicle control. ^#^P<0.05 vs. vehicle + 2-Me-5-HT. Graph A shows the summarized data and the insets represent the representative Western blot. B) Representative fluorescence images show the immunoreactivity for CaMKIIα (red) and pCaMKIIα (green) in EC cells treated as described in (A) and subjected to immunocytochemistry to determine 5HT_3_R-mediated CaMKIIα activation in isolated EC cells in vitro. Nuclei of EC cells were shown with DAPI stains. Scale bar, 4 µm.

The 5-HT-releasing EC cells present in the GIT are involved in the induction of emesis (see introduction). Furthermore, 2-Me-5-HT can activate 5-HT_3_Rs present on EC cells to promote release of endogenous serotonin from these cells and the induced release is sensitive to selective corresponding antagonists [3], [5], [6]. To investigate the direct actions of 2-Me-5-HT on EC cells, in this study we isolated EC cells from the least shrew GIT mucosa. The EC cells were incubated with either vehicle or palonosetron (1 µM) 30 min prior to addition of 2-Me-5-HT (1 µM), and cells were harvested at 20 min. The control group was exposed to vehicles of both palonosetron and 2-Me-5-HT in accord with the above described procedure. Western blots were performed on total proteins extracted from cell lysates to analyze the phosphorylation level of CaMKIIα at Thr286. The results showed significant increases in pCaMKIIα levels after 2-Me-5-HT exposure (P<0.05 vs. vehicle/vehicle control) (Figure 4A). Palonosetron pretreatment prevented the induced increase in CaMKIIα phosphorylation in response to 2-Me-5-HT (P<0.05 vs. vehicle + 2-Me-5-HT) (Figure 4A). Moreover, results obtained from immunoblots were confirmed using immunocytochemistry. The immunofluorescence of control EC cells showed weak immunoreactivity to CaMKIIα phophorylation at Thr286, which was elevated by 2-Me-5-HT incubation (Figure 4B). Pretreatment with palonosetron reversed the observed CaMKIIα activation evoked by 2-Me-5-HT (Figure 4B). These results provide evidence that 2-Me-5-HT directly increases CaMKIIα activation in vitro in EC cells via 5-HT_3_Rs.

### 5-HT_3_R-mediated vomiting occurs via Ca^2+^-dependent CaMKIIα activation

**Figure 5:**
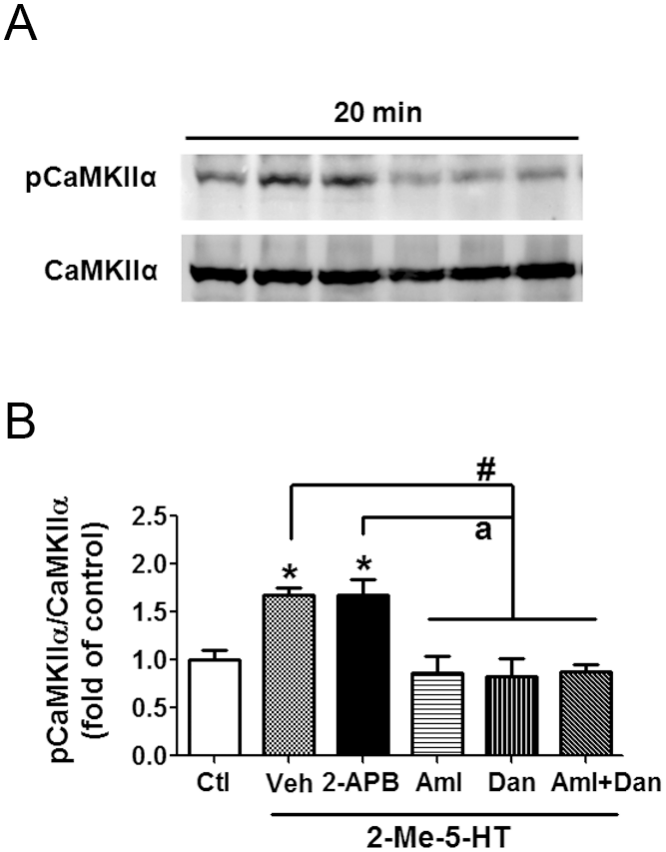
2-Me-5-HT-induced CaMKIIα activation is dependent upon Ca^2+^ mobilization mediated by L-type Ca^2+^ channels (LTCCs) and ryanodine receptors (RyRs). Different groups of shrews were administrated with either vehicle (Veh), or one the following agents, the LTCC blocker amlodipine (Aml, 10 mg/kg, s.c.), the RyR blocker dantrolene (Dan, 20 mg/kg, i.p.), a combination of less effective doses of amlodipine (5 mg/kg, s.c.) and dantrolene (10 mg/kg, i.p.) (Aml+Dan), or inositol-1, 4, 5-triphosphate receptor blocker 2-APB (10 mg/kg, i.p.), and 30 min later injected with 2-Me-5-HT (5 mg/kg, i.p.). Immunoblots were performed on brainstems of least shrews sacrificed 20 min after 2-Me-5-HT injection using anti-pCaMKIIα and CaMKIIα antibodies. n = 3 per group. The inset (A) shows the representative Western blot, and the graph (B) shows the fold change from individual experimental results. *P<0.05 vs. Veh/Veh control (Ctl). ^#^P<0.05 vs. Veh + 2-Me-5-HT. ^a^P<0.05 vs. 2-APB + 2-Me-5-HT).

**Figure 6:**
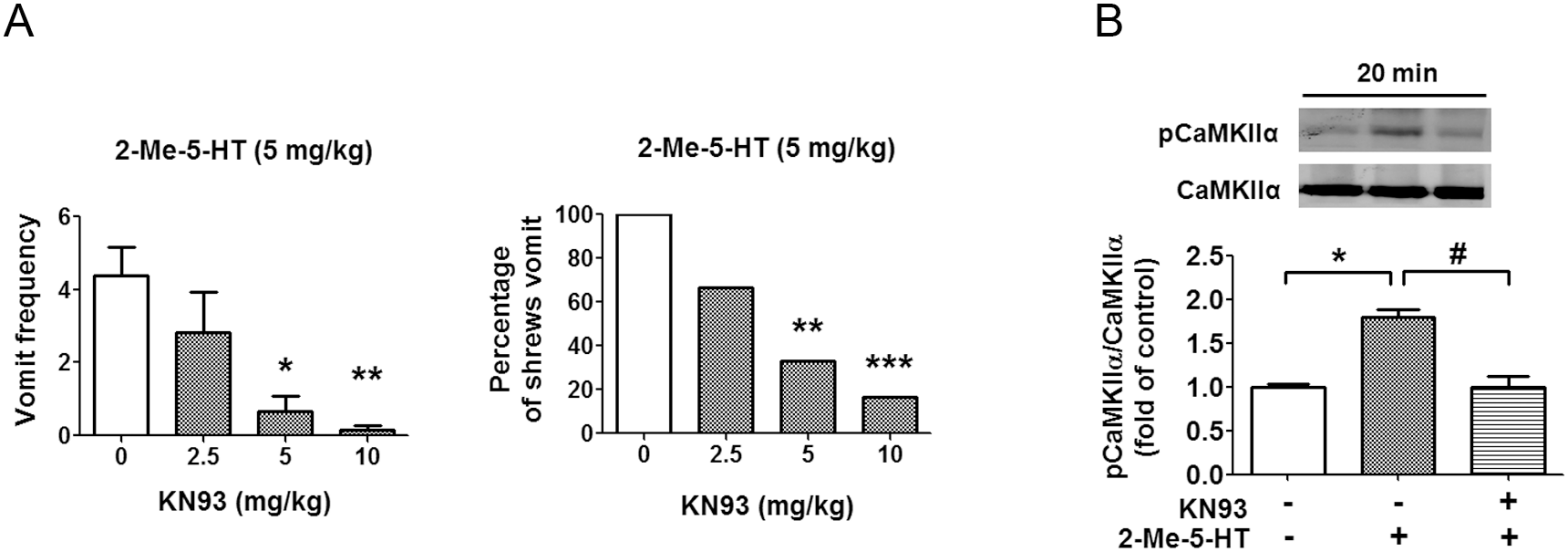
Effects of CaMKII inhibition on 5-HT_3_R-mediated emesis. A) The CaMKII inhibitor KN93 (i.p.) or its vehicle was administered to different groups of shrews 30 min prior to 2-Me-5-HT (5 mg/kg, i.p.) injection. The emetic responses were recorded for 30 min following 2-Me-5-HT injection. *P<0.05, **P<0.01 and ***P<0.001 vs. vehicle-pretreated control group. B) Immunoblot analyses of CaMKIIα phosphorylation were performed on brainstems collected from the experimental shrews 20 min after 2-Me-5-HT injection in the absence or presence of KN93 (10 mg/kg, i.p.). n = 3 per group. Graph B shows the fold change from individual experimental results and the insets demonstrate the representative Western blot. *P<0.05 vs. vehicle/vehicle control. ^#^P<0.05 vs. vehicle + 2-Me-5-HT.

In an effort to better understand the mechanisms involved in 5-HT_3_R-mediated CaMKIIα activation, we performed immunoblots on protein extracts of brainstems obtained from 2-Me-5-HT-treated shrews respectively pretreated with either amlodipine (10 mg/kg, s.c.), dantrolene (20 mg/kg, i.p.), or a combination of amlodpine (5 mg/kg, s.c.) and dantrolene (10 mg/kg, i.p.) (Figure 5A, B). In the behavioral result section of Figure 1, we demonstrated that each tested antagonist by itself possessed anti-emetic efficacy against 2-Me-5-HT-induced emesis, and a combination of their lower doses had greater antiemetic efficacy. Likewise, in the current experiment, we found that the 2-Me-5-HT-induced (*P<0.05) increase in pCaMKIIα immunoreactivity was significantly suppressed by the presence of amlodipine (10 mg/kg), dantrolene (20 mg/kg), or combined but lower doses of amlodipine (5 mg/kg) + dantrolene (10 mg/kg) (Figure 5B) (P<0.05 vs. vehicle + 2-Me-5-HT). However, 2-APB (10 mg/kg, i.p.) pretreatment failed to prevent the 2-Me-5-HT-evoked pCaMKIIα activation (P>0.05 vs. vehicle + 2-Me-5-HT) (Figure 5B). These results are in concordance with our described behavioral findings which suggest that elevation of intracellular Ca^2+^ via extracellular influx through L-type Ca^2+^ channels and intracellular Ca^2+^ release from ER Ca^2+^ stores through RyRs, but not IP_3_Rs, allow 2-Me-5-HT-induced CaMKIIα activation and emesis. To further validate a role for CaMKIIα activation in 5-HT_3_R-mediated emesis, we examined the antiemetic potential of the CaMKII inhibitor, KN93 (Figure 6). Thus, KN93 (0, 2.5, 5, 10 mg/kg, i.p.) was administered to different groups of shrews 30 min prior to 2-Me-5-HT (5 mg/kg, i.p.) injection. Relative to its vehicle-treated control group, KN93 pretreatment suppressed the frequency (>95%) of 2-Me-5-HT-induced vomiting (KW (3, 23) = 15.27, P<0.01), as well as the percentage of shrews vomiting (χ^2^ (3, 23) = 13.76, P<0.01) in a dose-dependent and potent manner (Figure 6A). In fact, significant reductions P<0.05–0.001) in these emetic parameters were seen at its 5 and 10 mg/kg doses. Moreover, an inactive analog of KN93 (i.e. KN92) at a dose of 10 mg/kg (i.p.), failed to suppress 2-Me-5-HT-induced vomiting (data not shown). The ability of KN93 to antagonize the 2-Me-5-HT-induced increase in pCaMKIIα in vivo was also analyzed by Western blots. The tested animals were sacrificed 20 min after 2-Me-5-HT treatment. As expected, KN93 pretreatment (10 mg/kg, i.p.) abolished the 2-Me-5-HT-induced CaMKIIα activation in brainstem (P<0.05, vehicle + 2-Me-5-HT vs. vehicle/vehicle control; P<0.05, KN93 + 2-Me-5-HT vs. vehicle + 2-Me-5-HT) (Figure 6B). These observations suggest that the Ca^2+^-modulated CaMKIIα activation in the brainstem is involved in 5-HT_3_R-mediated emesis.

### Activation of ERK1/2 by 5-HT_3_R stimulation in brainstem occurs through a Ca^2+^/CaMKII-dependent pathway

**Figure 7:**
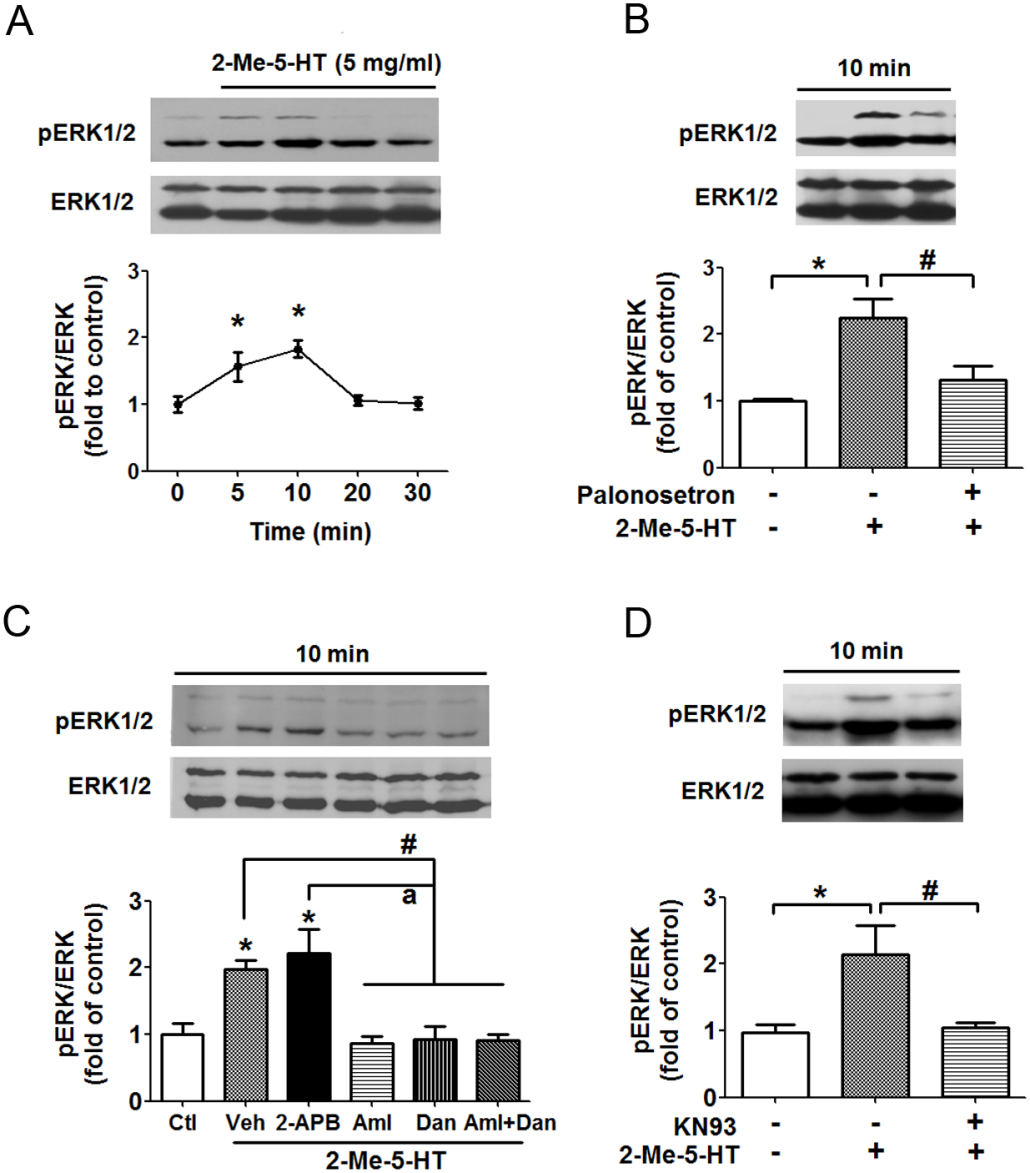
Involvement of Ca^2+^/CaMKIIα in 5-HT_3_R-mediated ERK activation. **A) Time-course of 2-Me-5-HT-induced ERK1/2 activation in the least shrew brainstem.** Least shrews were injected with 5 mg/kg (i.p.) 2-Me-5-HT and their brainstems were collected at 5, 10, 20 and 30 min (n = 3 per group). Phosphorylated (pERK1/2) and total ERK1/2 of the same sample from different shrews were determined by immunoblot with the antibodies to pERK1/2 and to total ERK1/2. The ratios of pERK1/2 (42 kD/44 kD) to ERK1/2 were calculated and expressed as fold change of vehicle-treated control (0 min). Graph A represents the summarized data and the insets show the representative Western blot. *P<0.05 vs. 0 min. Graphs B–D) Immunoblot analyses of ERK1/2 phosphorylation were performed on brainstems collected from the experimental shrews 10 min after 2-Me-5-HT treatment (5 mg/kg, i.p.) in the absence (vehicle) or presence of antagonists. B) Selective blockade of 5-HT_3_Rs with palonosetron (5 mg/kg, s.c.) 30 min prior to 2-Me-5-HT injection. *P<0.05 vs. vehicle/vehicle control and ^#^P<0.05 vs. vehicle + 2-Me-5-HT. C) Either vehicle (Veh, i.p.), the inositol-1, 4, 5-triphosphate receptor blocker 2-APB (10 mg/kg. i.p.), L-type Ca^2+^ channel blocker amlodipine (Aml, 10 mg/kg, s.c.), ryanodine receptor blocker dantrolene (Dan, 20 mg/kg, i.p.) or a combination (Aml+Dan) of less effective doses of amlodipine (5 mg/kg, s.c.) and dantrolene (10 mg/kg, i.p.) were administered to different groups of shrews 30 min prior to 2-Me-5-HT injection. *P<0.05 vs. Veh/Veh control (Ctl). ^#^P<0.05 vs. Veh + 2-Me-5-HT. ^a^P<0.05 vs. 2-APB + 2-Me-5-HT. D) Inhibition of CaMKII with KN93 (10 mg/kg, i.p.) blocked 2-Me-5-HT-evoked ERK1/2 phosphorylation in brainstem. n = 3 per group. Graphs show the summarized data and insets show representative Western blots. *P<0.05 vs. vehicle/vehicle control. ^#^P<0.05, vs. vehicle + 2-Me-5-HT.

It has been reported that CaMKII mediates ERK1/2 activation in response to Ca^2+^-mobilizing stimuli [34]. In the present study, we tested whether Ca^2+^/CaMKII regulates ERK1/2 signaling in response to 2-Me-5-HT administration (5 mg/kg, i.p.). Our attained time profile indicates that following 2-Me-5-HT administration, both pERK1 and pERK2 levels (pERK1/2) increased markedly in the least shrew brainstem at the 5 (P<0.05, vs. 0 min) and 10 min (P<0.05, vs. 0 min) exposure intervals, but returned towards baseline levels at 20 and 30 min (Figure **7**A). Therefore, a 10 min exposure time following 2-Me-5-HT injection was chosen to further investigate the role of Ca^2+^/CaMKII in ERK activation. No significant increase in ERK1/2 autophosphorylation occurred in response to 2-Me-5-HT treatment when shrews were pretreated with the 5-HT_3_R antagonist palonosetron (5 mg/kg, s.c.) (P>0.05, palonosetron + 2-Me-5-HT vs. vehicle/vehicle control) (Figure **7**B). This finding signifies that 5-HT_3_R stimulation mediates ERK1/2 signaling. Moreover, the 2-Me-5-HT-induced phosphorylation of ERK (Figure 7C, D; P<0.05) was also significantly suppressed via blockade of: i) extracellular Ca^2+^ influx through L-type plasma membrane Ca^2+^ channels with amlodipine (10 mg/kg; P<0.05); ii) intracellular Ca^2+^ release from ER stores through RyRs by dantrolene (20 mg/kg; P<0.05); iii) of both of these channels by lower but combined doses of amlodipine (5 mg/kg) and dantrolene (10 mg/kg) (P<0.05); or iv) CaMKII activity by its inhibitor KN93 (10 mg/kg) (P<0.05) (Figure **7**D). On the other hand, 2-APB pretreatment did not inhibit 2-Me-5-HT-evoked ERK1/2 activation (P>0.05 vs. vehicle + 2-Me-5-HT) (Figure 7C). Therefore, 5-HT_3_R-mediated ERK1/2 activation is a Ca^2+^/CaMKII-dependent process.

### Inhibition of ERK1/2 activation attenuates 2-Me-5-HT-induced vomiting

**Figure 8:**
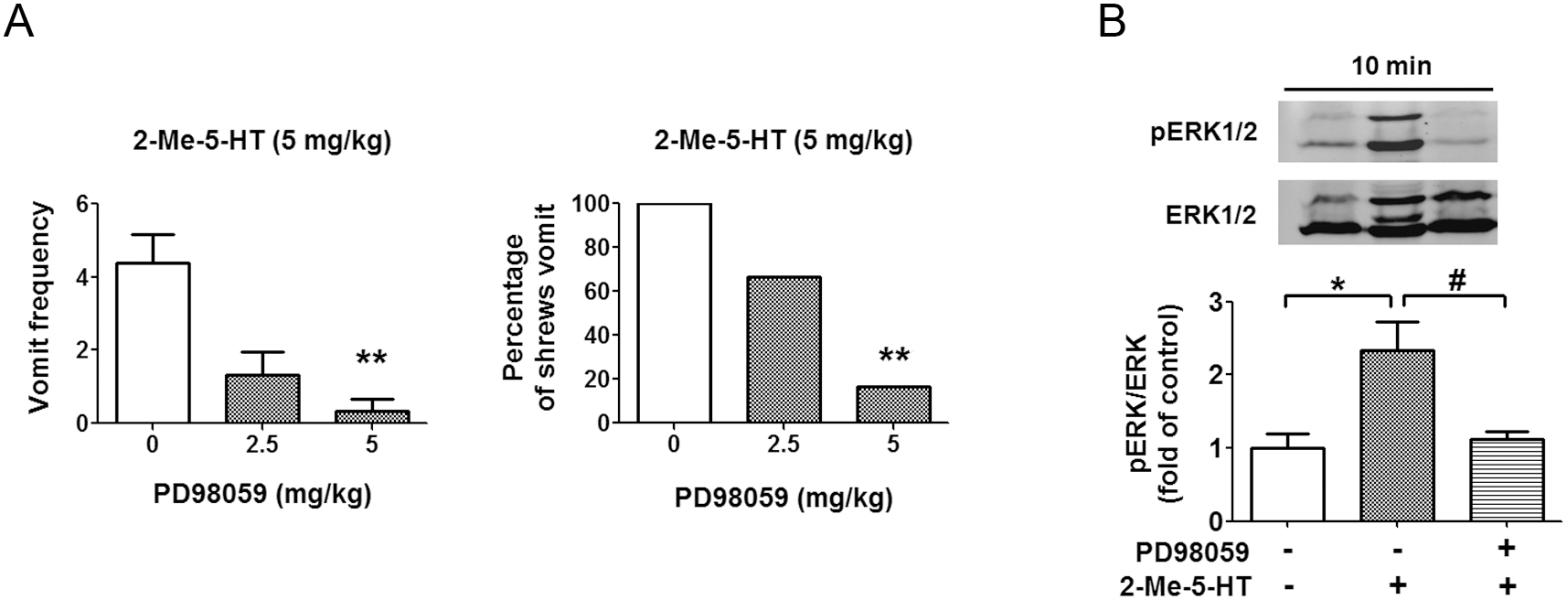
Suppressive effects of ERK inhibition on 5-HT_3_R-mediated emesis. A) The cited doses of the ERK inhibitor PD98509 were administered to different groups of shrews 30 min prior to 2-Me-5-HT (5 mg/kg, i.p.) injection. The vomit parameters were recorded for 30 min post 2-Me-5-HT injection. The vomit frequency data are presented as mean ± SEM. **P<0.01 and ***P<0.001 vs. vehicle-pretreated control. B) PD98059 (5 mg/kg, i.p.) or its vehicle (i.p.) was administered to different groups of shrews 30 min prior to 2-Me-5-HT (5 mg/kg, i.p.) injection and immunoblot analyses of ERK1/2 phosphorylation were performed on shrew brainstems collected 10 min after 2-Me-5-HT treatment. n = 3 per group. Graph B shows the summarized data and the insets show the representative Western blot. *P<0.05 vs. control vehicle/vehicle, ^#^P<0.05 vs. Vehicle + 2-Me-5-HT.

To test the anti-emetic potential of inhibition of ERK signaling, we pretreated least shrews with the ERK inhibitor PD98059 (0, 2.5 or 5 mg/kg, i.p.) 30 min prior to 2-Me-5-HT (5 mg/kg) injection. PD98059 reduced both the frequency (KW (2, 17) = 12.18, P<0.001) and percentage of shrews vomiting (χ^2^ (2, 17) = 10.48, P<0.01) in response to 2-Me-5-HT injection in a dose-dependent manner with ∼90% protection at 5 mg/kg (P<0.01) (Figure 8A). A 5 mg/kg dose of PD98059 also fully blocked (P<0.05, vs. vehicle + 2-Me-5-HT) the ability of 2-Me-5-HT to significantly activate ERK1/2 in the least shrew brainstem (P<0.05, vs. vehicle/vehicle control) (Figure 8B). Thus, 5-HT_3_Rs may use the ERK pathway to induce vomiting.

### 2-Me-5-HT-induced vomiting is independent of 5HT_2A_- and 5-HT_6_-receptor activity

**Figure 9:**
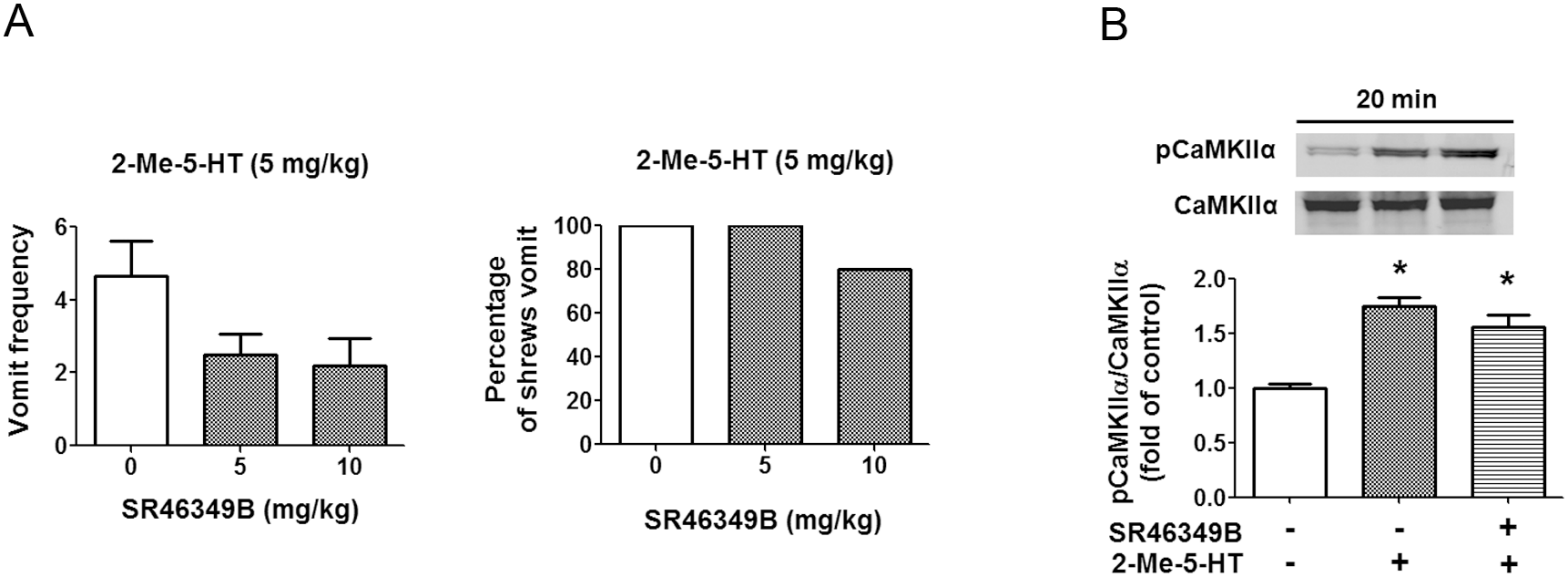
5-HT_2A_Rs antagonism has no significant effect on 2-Me-5-HT-evoked vomiting and CaMKIIα activation in the least shrew brainstem. A) Shrews were pretreated with the 5-HT2_A_R antagonist SR34649B (5, 10 mg/kg, s,c.) or vehicle 30 min prior to 2-Me-5-HT (5 mg/kg, i.p.) administration. The vomit parameters were recorded for 30 min post 2-Me-5-HT injection. B) Immunoblot analyses of CaMKIIα phosphorylation were performed on brainstems collected from the experimental shrews 20 min after 2-Me-5-HT treatment in the absence or presence of SR34649B (10 mg/kg, s.c.). n = 3 per group. Graph B shows the summarized data and the insets show the representative Western blot. *P<0.05 vs. control (vehicle/vehicle treated).

It has been suggested that functional interaction exists between 5-HT_2A_Rs and 5-HT_3_Rs [35]. To rule out the possibility that 5-HT_2A_Rs may be involved in emetic response evoked by 2-Me-5-HT, we evaluated the effect of 5-HT_2A/C_ R antagonist, SR46349B [36], [37]. Thus, SR46349B (5 and 10 mg/kg, s.c.) or its vehicle were administered to different groups of least shrews 30 min prior to 2-Me-5-HT. The vomiting response was recorded for the following 30 min. SR46349B (5 or 10 mg/kg) failed to significantly suppress either the frequency or the percentage of shrews vomiting in response to 2-Me-5-HT (Figure 9A). Western blots were further performed on brainstem protein extracts from least shrew pretreated with either SR46349B (10 mg/kg) or its vehicle 30 min prior to 2-Me-5-HT (5 mg/kg) injection. Tested animals were sacrificed at 20 min after 2-Me-5-HT injection. Consistent with the behavioral results, SR46349B had no significant effect (P>0.05) on the ability of 2-Me-5-HT to increase pCaMKIIα (Figure 9B). These findings strongly suggest that the 5-HT_3_R, and not the 5-HT_2A_R subtype, is specifically involved in 2-Me-5-HT-induced emesis-related responses.

In addition, 2-Me-5-HT has affinity for 5-HT_6_Rs [38] and consequently as described above we tested the antiemetic potentials of its corresponding antagonists (Ro-046790 [39] and Ro4368554 [40]) against the induced emesis. At doses 0.25, 1, 5, 10, and 20 mg/kg (i.p.) both agents failed to prevent the 2-Me-5-HT-evoked vomiting (data not shown). Thus, 5-HT_6_Rs are also not involved in vomiting.

## Discussion

**Figure 10:**
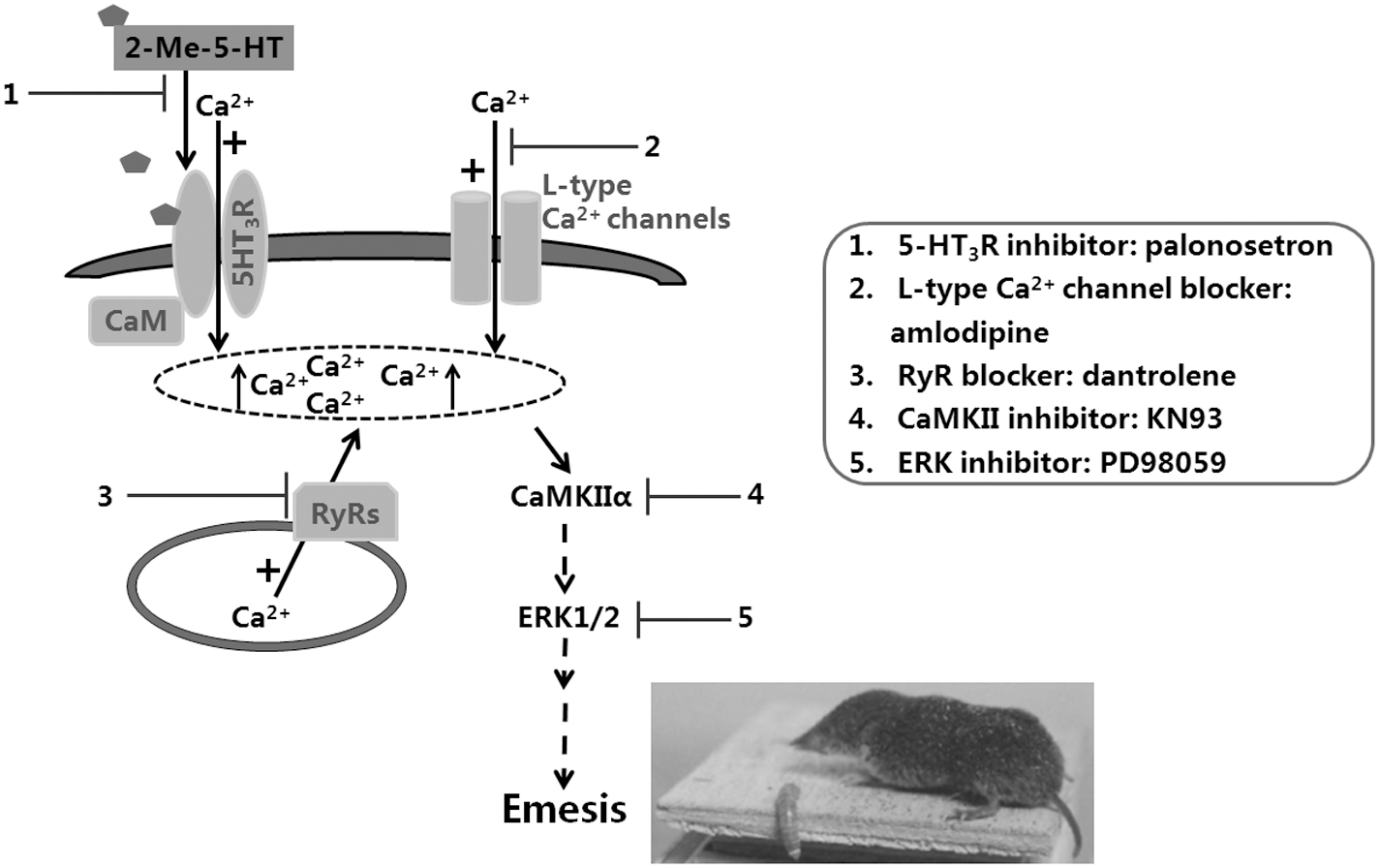
Summary of the proposed 5-HT_3_R-mediated downstream signaling pathway underlying 2-Me-5-HT-induced emesis in the least shrew. 5-HT_3_R stimulation by the selective agonist 2-Me-5-HT causes an influx of extracellular Ca^2+^ through 5-HT_3_Rs/L-type Ca^2+^ ion channels which increases the free cytoplasmic concentration of Ca^2+^, thereby promoting Ca^2+^ release via calcium-induced calcium release (CICR) from the endoplasmic reticulum stores through ryanodine receptors (RyRs). This elevation in cellular Ca^2+^ level initiates attachment of calmodulin (CaM) with the 5-HT_3_R, and leads to CaMKIIα activation and subsequent ERK1/2 signaling. The 5-HT_3_R antagonist palonosetron^(1)^, the L-type Ca^2+^ channel blocker amlodipine^(2)^, the RyR blocker dantrolene^(3)^, the CaMKII inhibitor KN93^(4)^, and the ERK inhibitor PD98059^(5)^, respectively exhibit anti-emetic efficacy against 2-Me-5-HT-induced vomiting. These findings demonstrate that the 2-Me-5-HT-induced emesis is regulated by 5-HT_3_R-mediated Ca^2+^/CaMKII-dependent ERK signaling pathway.

The concept and laboratory testing of antiemetic efficacy of 5-HT_3_R antagonists against CINV began in the early 1980s. To date, understanding of emetic signals downstream of 5-HT_3_R has remained elusive. Since chemotherapeutics such as cisplatin induce vomiting via concomitant release of several different emetogenic neurotransmitters [1], deciphering the downstream signal transduction mechanism(s) of a particular emetic transmitter in CINV becomes challenging, to say the least. Thus, in the current study we chose to investigate the post-receptor emetic signaling pathway of the more selective 5-HT_3_R “preferring” agonist 2-Me-5-HT in the least shrew. The advantage of this model over the long-standing and well-established ferret model is that unlike ferrets, shrews vomit consistently and in a dose-dependent manner in response to systemic administration of serotonin [11], [20]. Although serotonin cannot penetrate the blood-brain-barrier, its methylated analog, 2-Me-5-HT, does. We utilized pharmacological, behavioral, immunohistochemical, and Western blot techniques to reveal the central and peripheral emetic signaling components downstream of 5-HT_3_R activation in the induction of 2-Me-5-HT-evoked vomiting. Our findings support the hypothesis that, following 5-HT_3_R activation, 2-Me-5-HT causes an influx of extracellular Ca^2+^ through 5-HT_3_Rs/L-type Ca^2+^ channels, which subsequently evokes Ca^2+^-induced Ca^2+^ release (CICR) from intracellular ER Ca^2+^ stores via activation of RyRs Ca^2+^ channels present on the ER membrane. The enhanced Ca^2+^ mobilization is also sequentially linked to the intracellular activation of the CaMKIIα-ERK pathway in the brainstem, which plays an important role in 2-Me-5-HT-induced vomiting. (See our proposed signaling pathway in Figure 10).

### Involvement of extracellular Ca^2+^ influx and CICR in 5-HT_3_R-mediated emesis

Stimulation of 5-HT_3_Rs can increase intracellular Ca^2+^ levels via extracellular influx through both 5-HT_3_R- and voltage-dependent L-type Ca^2+^-channels present in the cell membrane [23], [41], [42], [43], [44]. In fact, the observed *in vitro* increase in Ca^2+^ influx into isolated cell lines is sensitive to both 5-HT_3_R- and L-type Ca^2+^ channel-selective antagonists [42], [43]. In the current *ex vivo* study we confirm that the selective 5-HT_3_R antagonist palonosetron can suppress the 5-HT_3_R-mediated, 2-Me-5-HT-evoked enhancements of intracellular Ca^2+^ concentration in the least shrew brainstem slices. Likewise, we have recently demonstrated that vomiting caused by specific stimulation of 5-HT_3_Rs in the least shrew is sensitive to selective antagonists of both 5-HT_3_Rs (e.g. palonosetron) and L-type Ca^2+^ channels (e.g. nifedipine) [15]. Moreover, the newly identified and novel emetogen FPL64176, a selective agonist of the L-type Ca^2+^ channels, causes vomiting in the least shrew in a dose-dependent manner. Not only palonosetron and nifedipine on their own can suppress FPL 64176-induced vomiting in a dose-dependent and potent manner, their ineffective but combined doses demonstrate significantly greater antiemetic efficacy against vomiting caused by several emetogens including FPL64176, 2-Me-5-HT and cisplatin [15]. These *in vivo* findings support the proposed cross-talk that occurs between 5-HT_3_Rs and L-type Ca^2+^ channels *in vitro* [45]. Consistent with these observations, in the current study we have demonstrated that vomiting triggered by 2-Me-5-HT is dose-dependently inhibited by another L-type Ca^2+^ channel blocker, amlodipine. Furthermore, both nifedipine and amlodipine are effective antiemetics against vomiting caused by diverse emetogens [15], [46].

Intracellular Ca^2+^ release from the endoplasmic reticulum (ER) can occur via at least two classes of receptors present in ER membrane termed IP_3_Rs and RyRs [47]. In addition, a functional linkage between L-type Ca^2+^ channels and RyRs appear to exist which plays an important role in intracellular Ca^2+^ release following voltage-dependent Ca^2+^ entry through L-type Ca^2+^channels [48], [49]. In the current study, we first determined whether 2-Me-5-HT-induced vomiting can be differentially modulated via manipulation of IP_3_Rs and RyRs. We found that the 5-HT_3_R-mediated vomiting was insensitive to the IP_3_R antagonist, 2-APB, but in contrast, was dose-dependently suppressed by the RyR antagonist, dantrolene. Furthermore, a combination of the semi-effective doses of amlodipine and dantrolene, was more potent than each antagonist being tested alone. These behavioral findings suggest that 5-HT_3_R stimulation drives extracellular Ca^2+^ through both 5-HT_3_Rs and L-type Ca^2+^ channels, which subsequently trigger Ca^2+^ release via RyRs from intracellular ER stores (i.e. CICR), which greatly amplifies free Ca^2+^ levels in the cytoplasm. Our *in vivo* findings are consistent with a previous *in vitro* cellular study which demonstrated that 5-HT_3_R activation evokes extracellular Ca^2+^ entry which then triggers such Ca^2+^ release from intracellular stores in a RyRs-sensitive manner (i.e. CICR) [42].

### Participation of CaM in 5-HT_3_R-mediated emesis

An increase in free cytoplasmic Ca^2+^ concentration can lead to activation of CaM and subsequent CaMKIIα [25]. The Ca^2+^ sensor CaM can regulate diverse functions by binding to hundreds of target proteins [50]. Our co-immunoprecipitation and immunohistochemistry findings provide the first evidence for an enhanced specific activity-dependent physical interaction between 5-HT_3_R and CaM in both the shrew brainstem and their colocalization in the jejunum following 2-Me-5-HT administration, since the observed association is sensitive to the 5-HT_3_R antagonist palonosetron. Indeed, it is already known that CaM can interact with several G-protein-coupled receptors including serotonergic 5-HT_1A_ [25]-, 5-HT_2A_ [26]-, and 5-HT_2C_ [27]-, as well as muscarinic M_1_-receptors [51], and alters their function via various means including desensitization, receptor internalization and trafficking. Therefore, our findings raise the possibility that in response to 5-HT_3_R activation by 2-Me-5-HT, CaM might influence the localization, clustering, and trafficking of 5HT_3_R as well as 5HT_3_R-mediated signal transduction via direct or indirect binding to 5-HT_3_R. Moreover, not only does CaM bind to L-type Ca^2+^ channels (LTCC) [52], [53] but our preliminary unpublished findings suggest that the discussed CaM-5-HT_3_R interaction can be also suppressed by the L-type Ca^2+^ channel antagonist, amlodipine. Thus, our findings suggest that 5-HT_3_R-CaM interaction appears to be regulated by 5-HT_3_R and LTCC activities which support the proposed crosstalk between 5-HT_3_R and L-type Ca^2+^ channels [45]. Based on our current report, the full role for CaM in the regulation of 5-HT_3_R signaling in general and in emesis in particular remains to be fully characterized, and more systematic experiments remain to be conducted, especially, (1) investigation of the consequences of 5-HT_3_R-CaM interaction on 5-HT_3_R function as an ion channel; and (2) cellular studies investigating the specific interruption of the 5-HT_3_R-CaM interaction with specific small molecules or peptides directly targeting the protein complexes as well as the influence of this specific blockade on 5-HT_3_R-mediated signaling pathway and emesis.

### 5-HT_3_R-mediated emesis occurs via Ca^2+^-dependent activation of CaMKIIα

CaMKII is a protein kinase that is widely expressed in a variety of tissues [54]. It autophosphorylates in response to elevated intracellular Ca^2+^ and functions as an intracellular signaling protein. Phosphorylated CaMKII (pCaMKII) has a relatively unique property that allows prolonged phosphorylation in response to transient Ca^2+^ signals making it an excellent marker for cellular activation. Furthermore, enhanced current through L-type voltage-gated Ca^2+^ channels can stimulate CaMKII activity which is required for various effects including induction of long-term potentiation [55], and cocaine-induced sensitization-specific adaptation of trafficking of GluA1 subunit of AMPA receptor [56]. Thus, a third novel aspect of this study was to determine whether Ca^2+^/CaMKII signaling is involved in the 5-HT_3_R-mediated 2-Me-5-HT-induced vomiting. In fact both vomit frequency and the degree of CaMKIIα activation appear to have a temporal relationship, since within 20 min of systemic injection, 2-Me-5-HT not only caused maximal number of vomits, but also induced maximal increase in CaMKIIα phosphorylation at Thr286 in brainstem as revealed by Western blots and immunohistochemistry. Similar to the reported differential increases in c-Fos immunoreactivity in the AP, NTS and DMNX of the least shrew in response to 2-Me-5-HT administration [14], CaMKIIα was also activated by 2-Me-5-HT in all of these brainstem DVC emetic nuclei, but the AP region exhibited higher activation. In addition, in the current study an identical pattern of results was obtained from isolated intestinal EC cells exposed to 2-Me-5-HT *in vitro*. Both Western blots of total protein extracted from least shrew EC cells and immunocytochemistry of EC cells exhibited substantial increases in pCaMKIIα levels. Moreover, pretreatment with the 5-HT_3_R antagonist palonosetron reversed the 2-Me-5-HT-induced increases in pCaMKIIα in the above-discussed *in vivo and in vitro* experiments. Since 5-HT_3_Rs are expressed in distinct cells in the GIT including functionally discrete classes of neurons as well as EC cells, 5-HT_3_R stimulation may involve the activation of both neuronal and nonneuronal pathways [5], [8]. In fact activation of 5-HT_3_Rs present on the surface of EC cells by 2-Me-5-HT can induce release of endogenous serotonin which can be prevented by prior exposure to selective 5-HT_3_R antagonists [5]. The released endogenous serotonin may then activate 5-HT_3_Rs on vagal nerve endings to initiate the vomiting reflex [6]. Thus, our current findings also appear to suggest the potential involvement of intracellular signaling mechanisms within EC cells in response to emetogens (2-Me-5-HT and possibly cisplatin or bacterial and viral toxins) for the release of endogenous serotonin in the mediation of emesis. In line with our above discussed findings, 5-HT release following perfusion of gut with glucose in rats has been shown to increase CaMKII phosphorylation in the EC cells, NTS and DMNX via activation of 5-HT_3_Rs [16]. Furthermore, 2-Me-5-HT-induced activation of CaMKIIα was abolished by prior treatment of least shrews with either the L-type Ca^2+^ channel antagonist amlodipine, the RyR antagonist dantrolene, or a combination of their less effective doses, but not by the IP_3_R antagonist 2-APB, which is consistent with the earlier discussed effects of these Ca^2+^ modulators on 2-Me-5-HT-induced vomiting presented in this study. In addition, the CaMKII inhibitor KN93 (but not its inactive analog KN92) [57] not only suppressed CaMKIIα phosphorylation in the shrew brainstem in response to 2-Me-5-HT, but also decreased the induced vomiting in a dose-dependent and potent manner. These results demonstrate that CaMKIIα activation contributes to 5-HT_3_R-mediated vomiting and is under regulation of extracellular Ca^2+^ influx through 5-HT_3_R/L-type Ca^2+^ channels as well as intracellular Ca^2+^ release from the ER stores via the RyRs.

### ERK signaling is necessary for 5-HT_3_R-induced emesis

We have recently demonstrated that significant activation of ERK1/2 is associated with peak vomit frequency during both the immediate and delayed phases of emesis caused by cisplatin in the least shrew [18]. In addition, serotonin plays an important role in both emetic phases in the brainstem and the GIT [9]. The final innovative finding of this study is that ERK1/2 activation in the brainstem occurs during 2-Me-5-HT-induced vomiting in the least shrew. This is also the first evidence that 5-HT_3_R stimulation is directly coupled to ERK1/2 phosphorylation. This upregulation of ERK1/2 was abolished by prior treatment with either palonosetron, amlodipine, dantrolene, KN93, or the ERK inhibitor PD98059, suggesting that extracellular Ca^2+^ influx, CICR from ER stores via RyRs, and CaMKII activation are sequential prior components of the ERK1/2 cascade involved in 5-HT_3_R-mediated signaling pathway. Our behavioral evidence that inhibition of ERK1/2 activation with PD98059 attenuated 2-Me-5-HT-induced emesis provides further credibility for the involvement of ERK1/2 in the induction of 5-HT_3_R-mediated emesis.

### 2-Me-5-HT-induced vomiting is independent of 5-HT_2A_R and 5-HT_6_R activation

Although 2-Me-5-HT is generally considered a 5-HT_3_R selective agonist, it does possess affinity for 5-HT_2A_Rs and 5-HT_6_Rs [58]. In fact 2-Me-5-HT administration in the least shrew can induce the protypical 5-HT_2A_ receptor-mediated head-twitch behavior [13]. Furthermore, 5-HT_2A_R stimulation can increase intracellular Ca^2+^ levels and affect L-type Ca^2+^ currents [59], [60]. In addition, functional interaction can occur between these two receptors where activation of 5-HT_2A_R potentiates 5-HT_3_R function [35]. In the current study we have demonstrated that the 5-HT_2A_R antagonist SR46349B, does not reduce the ability of 2-Me-5-HT to either induce vomiting or activate CaMKIIα in the shrew brainstem. Moreover, i.p. administration of the selective 5-HT_2A_R agonist, DOI, produces the head-twitch response in the least shrew [61] but not emesis [Darmani, unpublished observation]. Likewise, at diverse doses, we tested the antiemetic potential of two selective 5-HT_6_R antagonists (Ro-046790 and Ro-04368554). Both antagonists failed to suppress 2-Me-5-HT-evoked vomiting in the least shrew. Since we have recently demonstrated cAMP/PKA signaling is involved in mediation of cyclophosphamide-induced emesis [62], and activation of 5-HT_6_Rs can activate the cAMP/PKA cascade [63], we investigated the effect of 2-Me-5-HT on PKA phosphorylation at Thr197. 2-Me-5-HT had no significant effect on the latter parameter indicating that neither 5-HT_6_R nor its downstream signaling is involved in the induced vomiting (data not shown). Thus, the discussed findings strongly demonstrate that 5-HT_3_Rs (but not 5-HT_2A_Rs or 5-HT_6_Rs) are specifically involved in the mediation of 2-Me-5-HT-induced emesis and related downstream signaling.

## Conclusions

In summary, we postulate the following signaling pathway underlying 5-HT_3_R-mediated emesis: 2-Me-5-HT acts in both the brainstem DVC and the GIT emetic loci to increase extracellular influx of Ca^2+^ through both 5-HT_3_Rs and the L-type Ca^2+^ channels, which leads to CICR from intracellular ER calcium stores via RyRs. This 5-HT_3_R activation-induced increase in intracellular Ca^2+^ concentration initiates attachment of CaM to the 5-HT_3_R, and causes Ca^2+^-dependent activation of CaMKIIα which further results in ERK1/2 activation and vomiting (see Figure 10). The latter schematic provides new targets for the development of more novel antiemetics against diverse emetogens in general, and for those emetic agents (chemotherapeutics, bacterial and viral toxins) that employ 5-HT to induce vomiting, in particular.

## Supporting Information

Figure S1 **Effects of 2-Me-5-HT treatment on 5-HT_3_R-calmodulin (CaM) colocalization in the least shrew brainstem nucleus tractus solitaries (NTS) and dorsal motor nucleus of the vagus (DMNX).** Shrews were treated with 2-Me-5-HT (5 mg/kg, i.p.) or vehicle for 20 min. 5-HT_3_R-CaM colocalization was determined through co-stained brainstem slices with 5-HT_3_R (red) and CaM (green). Graphs A and B are representative images (200×) of NTS (A) and DMNX (B). Nuclei were shown with DAPI stains. Scale bar, 10 µm.
(TIF)

Figure S2 **Effects of 2-Me-5-HT treatment on pCaMKIIα in the least shrew brainstem nucleus tractus solitaries (NTS) and dorsal motor nucleus of the vagus (DMNX).** Shrews were treated with 2-Me-5-HT (5 mg/kg, i.p.) or vehicle for 20 min. CaMKIIα activation was determined through co-stained brainstem slices with CaMKIIα (red) and pCaMKIIα (green). Graphs A and B are representative images (100×) of NTS (A) and DMNX (B). Nuclei were shown with DAPI stains. Scale bar, 10 µm.
(TIF)
