# Supplementary figures and images for: Serotonin 5-HT3 Receptor-Mediated Vomiting Occurs via the Activation of Ca2+/CaMKII-Dependent ERK1/2 Signaling in the Least Shrew (Cryptotis parva)
Source: PLoS One. 2014 Aug 14;9(8):e104718. doi: 10.1371/journal.pone.0104718 (PMC4133232; doi:10.1371/journal.pone.0104718)

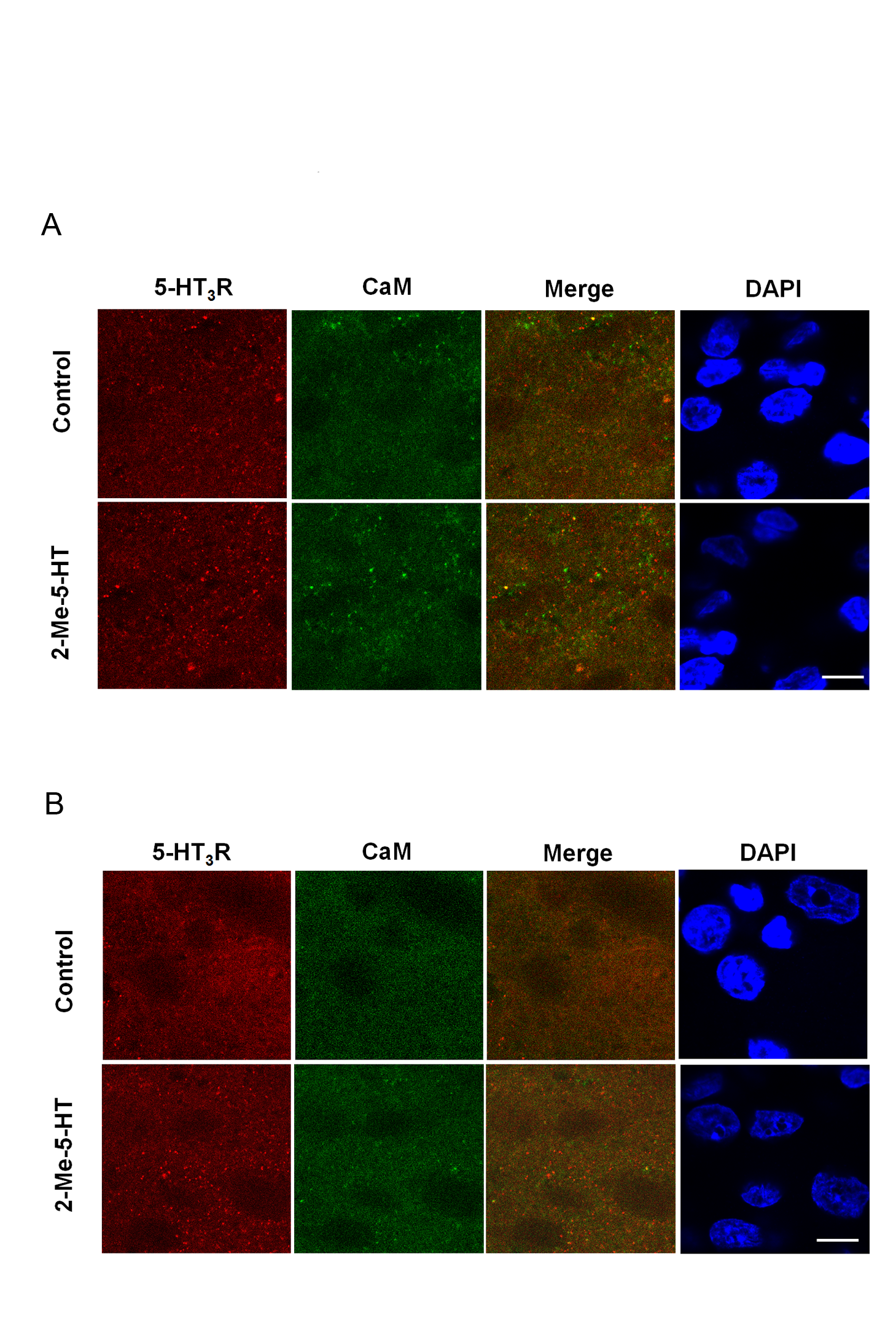

Supplement: Figure S1 — Effects of 2-Me-5-HT treatment on 5-HT3R-calmodulin (CaM) colocalization in the least shrew brainstem nucleus tractus solitaries (NTS) and dorsal motor nucleus of the vagus (DMNX). Shrews were treated with 2-Me-5-HT (5 mg/kg, i.p.) or vehicle for 20 min. 5-HT3R-CaM colocalization was determined through co-stained brainstem slices with 5-HT3R (red) and CaM (green). Graphs A and B are representative images (200×) of NTS (A) and DMNX (B). Nuclei were shown with DAPI stains. Scale bar, 10 µm. (TIF) [file pone.0104718.s001.tif]

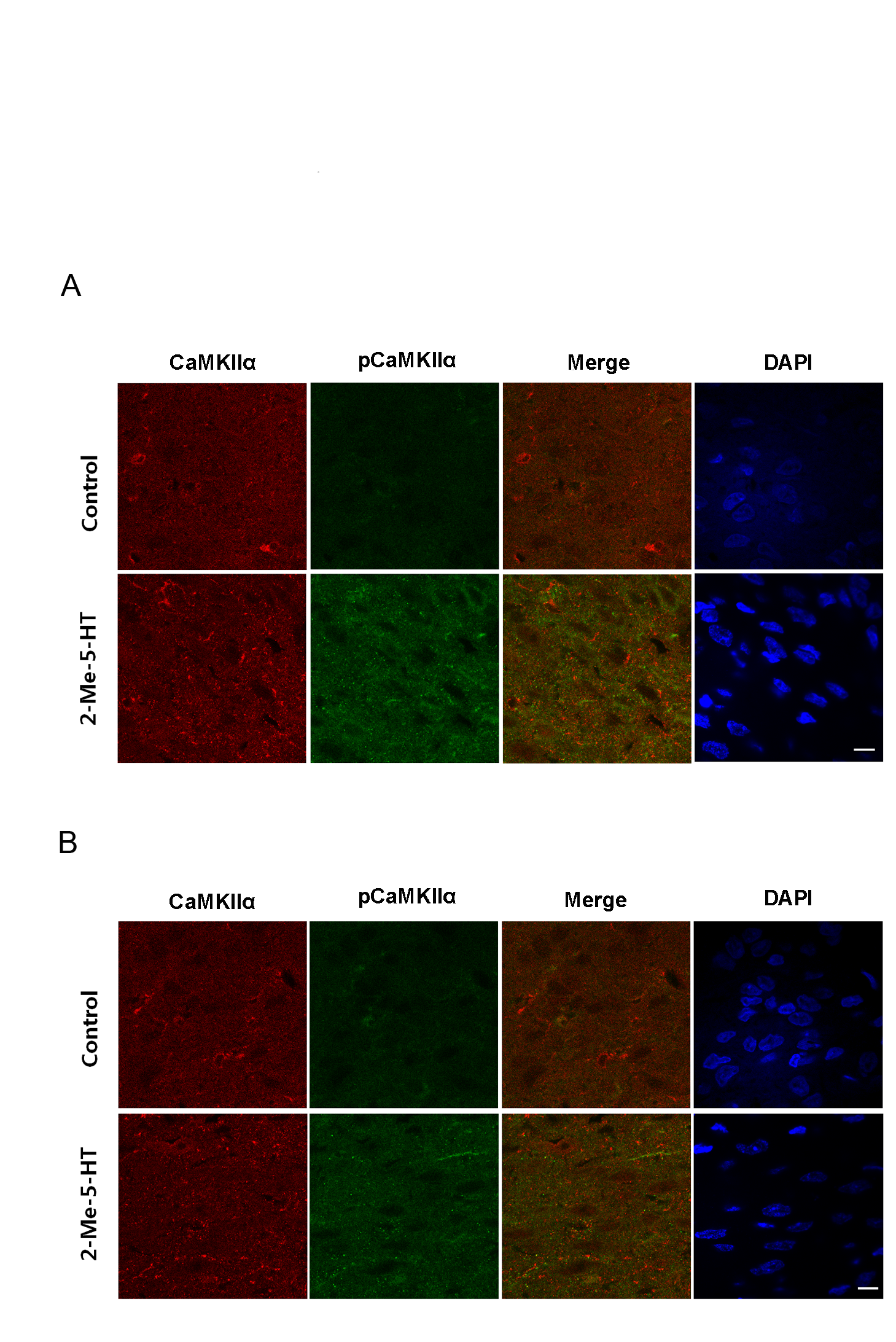

Supplement: Figure S2 — Effects of 2-Me-5-HT treatment on pCaMKIIα in the least shrew brainstem nucleus tractus solitaries (NTS) and dorsal motor nucleus of the vagus (DMNX). Shrews were treated with 2-Me-5-HT (5 mg/kg, i.p.) or vehicle for 20 min. CaMKIIα activation was determined through co-stained brainstem slices with CaMKIIα (red) and pCaMKIIα (green). Graphs A and B are representative images (100×) of NTS (A) and DMNX (B). Nuclei were shown with DAPI stains. Scale bar, 10 µm. (TIF) [file pone.0104718.s002.tif]
